# Maternal mortality, stillbirths, and neonatal mortality: a transition model based on analyses of 151 countries

**DOI:** 10.1016/S2214-109X(23)00195-X

**Published:** 2023-06-20

**Authors:** Ties Boerma, Oona M R Campbell, Agbessi Amouzou, Cauane Blumenberg, Hannah Blencowe, Allisyn Moran, Joy E Lawn, Gloria Ikilezi

**Affiliations:** a Institute for Global Public Health and Department of Community Health Sciences, University of Manitoba, Winnipeg, MB, Canada; b Department of Infectious Disease Epidemiology, London School of Hygiene & Tropical Medicine, London, UK; c Department of International Health, Johns Hopkins University, Baltimore, MA, USA; d Centre for Equity in Health, Federal University of Pelotas, Pelotas, Brazil; e Maternal Health Unit, WHO, Geneva, Switzerland; f Exemplars in Global Health, Gates Ventures, Seattle, WA, USA

## Abstract

**Background:**

Maternal mortality, stillbirths, and neonatal mortality account for almost 5 million deaths a year and are often analysed separately, despite having overlapping causes and interventions. We propose a comprehensive five-phase mortality transition model to improve analyses of progress and inform strategic planning.

**Methods:**

In this empirical data-driven study to develop a model transition, we used UN estimates for 151 countries to assess changes in maternal mortality, stillbirths, and neonatal deaths. On the basis of ratios of maternal to stillbirth and neonatal mortality, we identified five phases of transition, in which phase 1 has the highest mortality and phase 5 has the lowest. We used global databases to examine phase-specific characteristics during 2000–20 for causes of death, fertility rates, abortion policies, health workforce and financing, and socioeconomic indicators. We analysed 326 national surveys to assess service coverage and inequalities by transition phase.

**Findings:**

Among 116 countries in phases 1 to 4 in 2000, 73 (63%) progressed at least one phase by 2020, six advanced two phases, and three regressed. The ratio of stillbirth and neonatal deaths to maternal deaths increased from less than 10 in phase 1 to well over 50 in phase 4 and phase 5. Progression was associated with a declining proportion of deaths caused by infectious diseases and peripartum complications, declining total and adolescent fertility rates, changes in health-workforce densities and skills mix (ie, ratio of nurses or midwives to physicians) from phase 3 onwards, increasing per-capita health spending, and reducing shares of out-of-pocket health expenditures. From phase 1 to 5, the median coverage of first antenatal care visits increased from 66% to 98%, four or more antenatal care visits from 44% to 94%, institutional births from 36% to 99%, and caesarean section rates from 2% to 25%. The transition out of high-mortality phases involved a major increase in institutional births, primarily in lower-level health facilities, whereas subsequent progress was characterised by rapid increases in hospital births. Wealth-related inequalities reduced strongly for institutional birth coverage from phase 3 onwards.

**Interpretation:**

The five-phase maternal mortality, stillbirth, and neonatal mortality transition model can be used to benchmark the current indicators in comparison to typical patterns in the transition at national or sub-national level, identify outliers to better assess drivers of progress, and inform strategic planning and investments towards Sustainable Development Goal targets. It can also facilitate programming for integrated strategies to end preventable maternal mortality and neonatal mortality and stillbirths.

**Funding:**

Bill & Melinda Gates Foundation.

## Introduction

Maternal mortality, stillbirths, and neonatal mortality are major global health issues with an estimated 0·3 million maternal deaths, 1·9 million stillbirths, and 2·4 million neonatal deaths in 2020.^1^, ^2^, ^3^ In 1985, the neglect of maternal mortality within maternal and child health strategies was recognised and stimulated the global safe-motherhood initiative.^4^ Neonatal mortality gained more prominence about two decades later, when mortality in children younger than 5 years fell rapidly, but the proportion of neonatal deaths among these deaths increased.^5^ Stillbirths remain neglected to this day in maternal and neonatal health programmes.^6^ These three target groups each need specific attention, but integrated and synergistic approaches are also important.^7^ Yet, they are usually analysed as separate entities in global health. Mortality determinants are interconnected for pregnant women and their babies, as are underlying health conditions, interventions, and service-delivery platforms. An integrated mortality-transition model is currently lacking.

Transition models have previously been used to portray changes in other population and health outcomes. The demographic transition model from high to low mortality and fertility is characterised by a mortality decline preceding a fertility decline, resulting in a period of substantial population growth and major changes in population age structure.^8^, ^9^ The epidemiological transition represents changes in the causes of mortality and morbidity from a predominance of acute communicable to chronic non-communicable conditions, while all-cause mortality declines.^10^, ^11^ Both transition models segment changes over time into phases or stages.^12^

**Research in context**

**Evidence before this study**

Maternal mortality, stillbirth rates, and neonatal mortality have been declining rapidly in the second half of the 20th century and most prominently during the past two to three decades. This Article fills a crucial gap in our ability to understand the drivers of past progress, analyse the current situation, and to develop more effective strategies in the context of the 2030 Sustainable Development Goals. Building upon the demographic and epidemiological transition theories, and previous work on an obstetric transition and neonatal mortality declines, we developed a comprehensive five-phase transition model for maternal, stillbirth and neonatal mortality, considering several characteristics including mortality patterns, causes of death, fertility, abortion policies, health systems, socioeconomic progress, health service coverage, and inequalities. We quantified phase-specific patterns as countries progress from high to low mortality, using national data from 151 countries, historical data, and more than 300 household surveys from low-income and middle-income countries since 2000.

**Added value of this study**

This study is, to our knowledge, the first to integrate knowledge and evidence on drivers of the maternal, stillbirth, and neonatal mortality trends in a single transition model. For each of the five phases of the high-to-low maternal, stillbirth, and neonatal mortality transition, we identified common characteristics for the multiple dimensions, such as the role of infectious diseases as a cause of death, overall and adolescent fertility rates, health workforce density, coverage of births by all health facilities and by hospitals, and inequalities in service coverage and caesarean section. We provided typical values for each phase, which allow benchmarking of the current situation of a given country against common patterns on the basis of the experience of other countries at the same transition phase.

**Implications of all the available evidence**

The approach provides a systematic tool to better understand key characteristics of progress during the past few decades and to inform strategic planning by comparing current indicators with common patterns in the subsequent transition phases. It also presents a model for better integration of maternal and neonatal health programmes, including stillbirths.

In the field of maternal health, an obstetric transition model has been used to understand maternal mortality reduction across countries.^13^, ^14^ This model includes four phases with mortality thresholds of 1000, 300, and 50 maternal deaths per 100 000 livebirths. Lawn and colleagues^5^ used a similar approach to classify countries by neonatal mortality thresholds of 45, 30, 15, and five per 1000 livebirths, to identify differences in fertility, causes of death, and service-coverage indicators, although they did not describe their work as a transition model.^15^, ^16^, ^17^

We developed a combined model for a maternal, stillbirth, and neonatal mortality transition with five phases, and assessed how causes of death, fertility, abortion policies, health-system characteristics, service coverage, and inequalities changed between phases, and how much heterogeneity there was within phases. The transition model aims to facilitate further integration of maternal, stillbirth, and neonatal mortality analyses, provide a tool for benchmarking country progress, improve understanding of past mortality change and its drivers, and inform strategic planning and programming in specific countries and globally.

## Methods

### Data

In this empirical data-driven study to develop a mortality transition model, we combined the neonatal mortality rate (death 0–27 days after birth) with stillbirths (late-gestation fetal deaths from 28 weeks of pregnancy, as per the WHO definition for international comparisons; appendix p 1) using the same denominator of total births (ie, livebirths and stillbirths) into one measure (stillbirth and neonatal mortality). Maternal deaths, expressed per 100 000 livebirths in line with current practice, were kept separate because maternal deaths are several orders of magnitude rarer.

Our main analysis focused on national estimates for 151 countries with a population of at least 1 million in 2000. We used UN estimates for 2000–20 for maternal mortality,^1^ stillbirths,^2^ and neonatal mortality.^3^ Data on causes of death, fertility rates, abortion policies, health workforce, health financing, and socioeconomic indicators were extracted from WHO, the UN Population Division, and World Bank databases. We have provided details of the data sources in the appendix (pp 1–2). For service coverage indicators, including inequalities, and neonatal mortality rates by place of birth, we analysed data from 326 national Demographic and Health Surveys and Multiple Indicator Cluster Surveys conducted during 2000–20. We examined historical data with time series on maternal, stillbirth, and neonatal mortality from countries in western Europe, North America, and Asia, as well as prospective studies on the outcomes of pregnancy in higher-mortality settings in south Asia and Africa (appendix pp 3–8).

### Mortality thresholds

We analysed trends in the rate ratios of stillbirths to neonatal mortality, and of maternal to the sum of stillbirth and neonatal mortality (stillbirth plus neonatal deaths). Stillbirth rates and neonatal mortality are highly correlated in historical data, in prospective studies of pregnancy outcomes, and in global estimates (appendix pp 3–8). The ratio of stillbirths to neonatal deaths generally ranged from 0·7 to 1·1 and was weakly correlated with levels of mortality, except at low levels of mortality when stillbirths became more prominent. We combined stillbirths and neonatal deaths into one measure (stillbirths plus neonatal deaths per 1000 births).

Maternal mortality ratios are highly correlated with stillbirth^18^ and neonatal mortality rates. The ratio of stillbirth and neonatal deaths to maternal deaths increased from less than 20 to more than 75 as mortality declined in the historical data and the UN estimates (appendix pp 3–14). In prospective studies of pregnancy outcomes, the median ratio was 27 (IQR 17–33) and the ratios did not vary systematically by mortality levels.^19^, ^20^

Using thresholds from a published obstetric transition model as a starting point,^13^ we reviewed historical data on maternal mortality from high-income countries. By the 1930s, maternal mortality differed greatly between countries, from about 300 per 100 000 livebirths in Scandinavian countries and the Netherlands to 500 in England and Wales and 700 in the USA.^21^, ^22^, ^23^ Historical trends from Malaysia and Sri Lanka, both heralded as success stories in maternal health, support these thresholds.^24^ We selected 700, 300, 100, and 20 maternal deaths per 100 000 livebirths as thresholds for maternal mortality, in which the lowest value indicated that a population was approaching elimination of all preventable maternal deaths. For stillbirth and neonatal mortality, we used previously published neonatal mortality thresholds of 45, 30, 15, and five deaths per 1000 livebirths, to reach stillbirth plus neonatal mortality thresholds of 80, 55, 30, and 15 or fewer per 1000 births, respectively.

We combined these mortality thresholds into a five-phase transition model in which phase 1 was characterised by the highest maternal mortality (≥700 per 100 000 livebirths) and stillbirth plus neonatal mortality (≥80 per 1000 births) and phase 5 by the lowest maternal (<20 per 100 000 livebirths) and stillbirth plus neonatal (<15 per 1000 births) mortality. The ratios of stillbirth plus neonatal mortality to maternal mortality increased from 11 to 18, 30, and 75 in the phase 1 to 5 transition knots; for instance, the transition knot from phase 3 to 4 is characterised by stillbirth plus neonatal mortality of 55 per 1000 births and maternal mortality of 300 per 100 000 livebirths, which translates into a ratio of 18. A country was considered to have reached the next transition phase only when both mortality indicators passed the required thresholds.

### Phase characteristics

As the transition progresses, changes in cause of death, fertility, health systems, service coverage, and inequalities in coverage can be expected. We examined these changes using several datasets of global estimates and household surveys.

A common cause-of-death structure is part of an integrated transition model. The main causes of maternal death,^25^ stillbirth,^26^ and neonatal death^27^ were combined into three broad groups, which comprised infectious diseases (group 1, including abortion complications), causes related to the health and nutritional status of the woman or baby (group 2, including indirect causes for maternal deaths, prematurity, and intrauterine growth restrictions), and peripartum complications (group 3; appendix pp 15–19). In historical data, the transition was characterised by declining importance of infectious diseases and, to a lesser extent, group 3 causes, whereas group 2 causes became more prominent.^23^, ^24^, ^28^

Regarding fertility, the demographic transition posits that the all-cause mortality decline precedes fertility decline. Historical data from Sri Lanka and Malaysia indicated that maternal and neonatal mortality were already declining before the onset of fertility decline.^24^ A mutually reinforcing effect of mortality and fertility declines is probable, because fertility influences maternal, stillbirth, and neonatal mortality risks by changes in the age, parity, and birth interval distribution.^29^ Fewer children might also contribute to increasing service coverage and quality, and have a generational effect on the health and nutrition of women. We analysed long-term trends of fertility and neonatal mortality from 1970 onward. Such trends were not available for stillbirths or maternal mortality trends in most countries. For abortion policies, we generated a score on the basis of five legal grounds for abortion, in which the lowest score indicated that abortion was not legally permitted in any circumstances and the highest score that abortion is available on request (appendix pp 20–21).

To assess changes in health systems, we analysed phase-specific changes in total health expenditure per capita, total health expenditure as a percentage of gross domestic product (GDP), and out-of-pocket expenditure as a percentage of total health expenditure, as well as density of core health professionals (physicians and nurses, midwives, or nurse-midwives [as applicable]) and the skills-mix ratio (ie, the ratio of nurses and midwives to physicians).

Historical data provided evidence of the association between coverage of institutional delivery care and mortality.^21^, ^22^, ^23^, ^24^, ^30^ We examined the trends in antenatal care visits (one or more visits, and four or more visits), institutional livebirths, and caesarean section by phase on the basis of the household surveys. For institutional livebirths, we further analysed coverage mortality by place of birth (hospital; health centres or smaller health facilities [referred to as lower-level facilities]; and home).

Empirical data on social inequalities in the timing and pace of the epidemiological transition within countries have shown considerable heterogeneity.^31^, ^32^, ^33^, ^34^ We assessed inequalities in institutional livebirth coverage by wealth quintiles, focusing on the absolute gaps and the wealth-related inequality patterns by phase. We used the inequality patterns index, defined as the difference in the gap between the bottom and top quintiles compared with the national mean, to assess the presence of top inequalities (ie, when individuals with the highest wealth have much higher coverage than all other wealth quintiles) or bottom inequalities (ie, when individuals with the lowest wealth have much lower coverage than all other wealth quintiles).^35^ In addition, we analysed caesarean sections per 1000 livebirths among the poorest and richest wealth quintiles by phase. Finally, we assessed the extent to which socioeconomic changes occur concurrently with the mortality transition using per-capita income and female education levels.

All analyses were done in Stata 17.0 and Microsoft Excel 365. Because all data used in this study are in the public domain, no ethical clearance was required.

### Role of the funding source

The funder of this study had no role in study design, data collection, data analysis, data interpretation, or writing of the report.

## Results

**Figure 1:**
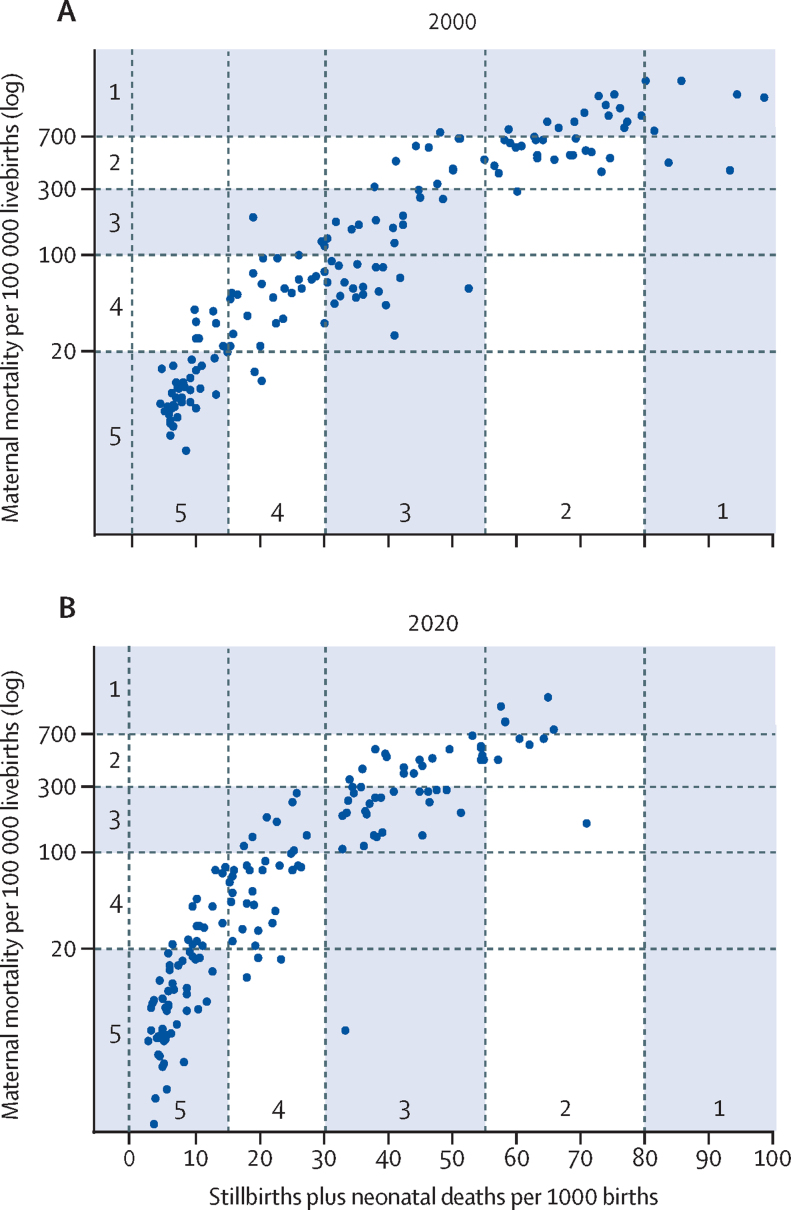
Maternal mortality per 100 000 livebirths and stillbirth plus neonatal mortality per 1000 births Data are based on UN mortality estimates for 151 countries in 2000 (A) and 2020 (B).

**Figure 2:**
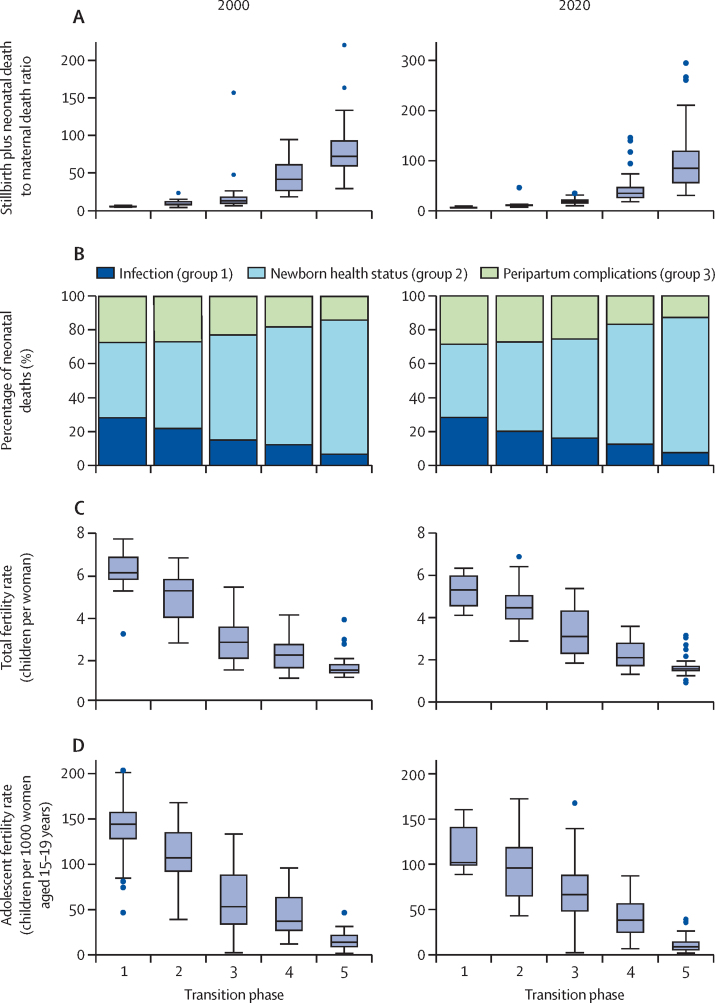
Selected mortality characteristics by mortality transition phase in 151 countries in 2000 and 2020 Cause of death estimates are for 2000 and 2019.

In 2000, based on the UN country mortality estimates, 21 (14%) of the 151 countries were in phase 1, of which 18 were in sub-Saharan Africa, 29 (19%) were in phase 2, 34 (23%) in phase 3, 32 (21%) in phase 4, and 35 (23%) in phase 5 (figure 1). By 2020, five (3%) countries were in phase 1 (Chad, Central African Republic, Nigeria, Sierra Leone, and Somalia), 23 (15%) were in phase 2, 32 (21%) in phase 3, 42 (28%) in phase 4, and 49 (32%) in phase 5. The ratio of stillbirth plus neonatal deaths to maternal deaths increased from 10 or less in phase 1 more than 50 in phase 4 and more than 70 in phase 5 (figure 2A).

Among the 116 countries in phases 1 to 5 in 2000, 73 (63%) progressed at least one phase during 2000–20. Six countries progressed two phases: Angola, Bangladesh, Ethiopia, Kazakhstan, Rwanda, and Tanzania. Three countries—the USA, Venezuela, and Viet Nam—regressed one phase, all because of increases in maternal mortality (country-specific data are shown in appendix p 14).

We also classified countries on the basis of the lower and upper bounds of the uncertainty ranges of the mortality estimates. This classification resulted in markedly different distributions of countries by phase, with a mean difference of 0·5 phases between the lower and upper bounds in 2000 and 0·6 phases between the lower and upper bounds in 2020 (appendix pp 9–13).

Among neonates, cause-of-death distributions differed by transition phase: group 2 causes took a greater share of the distribution (from 41% in phase 1 to 72% in phase 5), whereas group 1 (from 24% in phase 1 to 6% in phase 5) and group 3 (from 26% in phase 1 to 13% in phase 5) took a smaller share of the distribution (figure 2B). For maternal mortality, only a modest decrease in the relative importance of group 1 causes and group 3 peripartum causes was observed in global estimates (appendix pp 15–19).

There was a strong association between total fertility rate and transition phase. The country median for total fertility rate in 2000 was 6·1 children per woman in phase 1 (IQR 5·8–6·9), declining to 5·4 (4·0–5·8) in phase 2, 2·8 (2·0–3·5) in phase 3, 2·2 (1·6–2·7) in phase 4, and 1·5 (1·4–1·8) in phase 5. Fertility by transition phase in 2020 showed a similar pattern, declining from 5·3 (4·5–6·0) in phase 1 to 1·5 (1·4–1·8) children per woman in phase 5 (figure 2C). Adolescent fertility also declined from more than 100 births per 1000 women aged 15–19 years in phase 1 and 2, to 60 in phase 3, 40 in phase 4, and about 15 in phase 5, in both 2000 and 2020 (figure 2D). In low-income and middle-income countries with neonatal mortality and fertility trend data available before 1980, neonatal mortality declines either preceded or ran in parallel to the fertility declines (appendix pp 20–21).

The abortion policy score was lowest (most restrictive) in phase 1 and highest (most permissive) in phase 5, gradually increasing from scores of 51% (2000) and 40% (2020) in phase 1 to 68% (2000) and 59% (2020) in phase 3 and 86% (2000) and 91% (2020) in phase 5 (appendix p 21).

Both gross national income per capita and female education levels, measured by gross female secondary enrolment, increased strongly by phase in 2000 and 2020 (appendix pp 22–23). Income increased most prominently from phase 3. Secondary enrolment among girls increased substantially during the first three phases, doubling from phase 1 to phase 2 and again from phase 2 to phase 3, reaching 75%.

**Figure 3:**
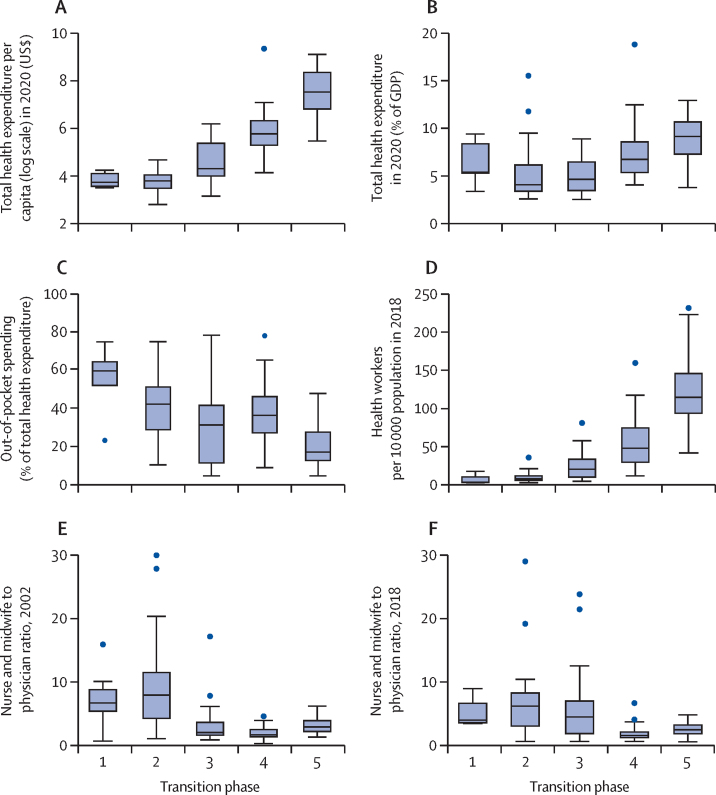
Health financing and health workforce indicators by mortality transition phase in 138 countries In (E), Liberia's ratio was 38·5, but was scaled down to 29 in this graph for presentation purposes. GDP=gross domestic product.

Total health expenditure per capita in 2020 was less than US$45 in the first two phases and then nearly doubled in countries in phase 3 and again tripled in phase 5 to more than $300 per capita (figure 3A). Government spending on health remained 4–5% of GDP in the first three phases and increased in phase 4 to nearly 7% (figure 3B). Out-of-pocket spending was higher in the early phases but the large variability between countries in phases 2 to 4 was notable (figure 3C; appendix pp 24–26).

Median density of core health professionals was low in the first two phases (four per 10 000 population in phase 1 and eight per 10 000 in phase 2), increasing to 20 per 10 000 in phase 3 and 43 per 10 000 in phase 4 (figure 3D). The first two phases of the transition were associated with much higher ratios of nurses or midwives to physicians than in later phases. The ratio peaked in phase 2 at 8 nurses or midwives to a physician in 2002 and 6 in 2018 (figure 3E, 3F). In phase 4, the median was less than 2 nurse or midwives to a physician in both years. Countries in phase 3 had a median ratio of 2 nurses or midwives to a physician in 2002 and 4·5 in 2018, showing that in 2018 more countries progressed into phase 3 with greater reliance on nurses and midwives than physicians than in 2002.

**Figure 4:**
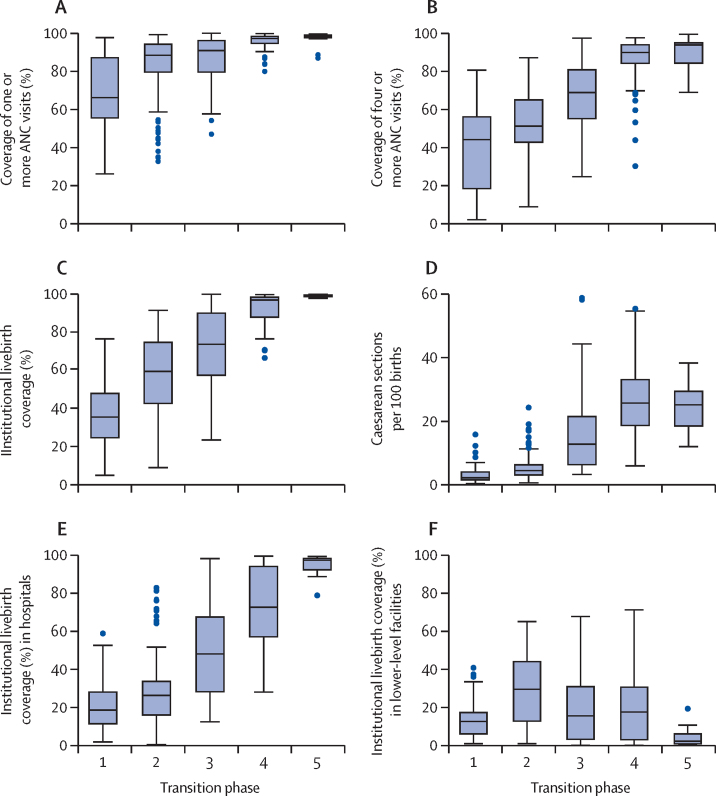
Coverage of antenatal and delivery care, by maternal, stillbirth, and neonatal mortality transition phase Data are results from national surveys, 2000–20. ANC=antenatal care visit.

On the basis of surveys during 2000–20, median coverage of one or more antenatal care visits increased from 66% to 98%, of four or more antenatal care visits from 44% to 94%, institutional birth coverage from 36% to 99%, and caesarean section rates from 2% to 25% across phase 1 to 5 (figure 4A–D). Rapid increases occurred early in the transition (one or more antenatal care visits), throughout from phase 1–5 (institutional births), and in the middle phases (four or more antenatal care visits and caesarean sections; appendix pp 27–28).

Hospital births became more common as coverage increased across phases (figure 4E, F). The transition from phase 1 to 2 primarily involved a major increase of births in lower-level health facilities. The progression from phase 3 into phases 4 and 5 was associated with major increases in hospital births to near universality (appendix p 27).

**Figure 5:**
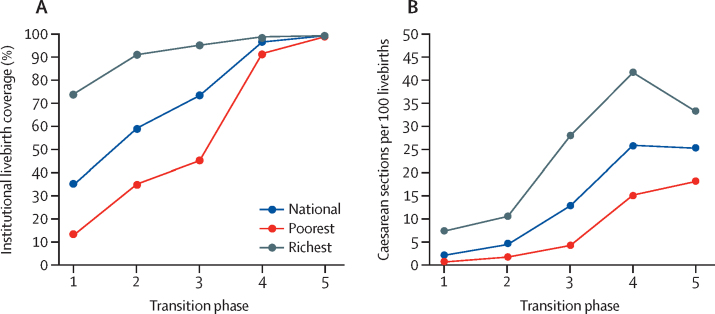
Coverage of institutional livebirths and caesarean sections per 100 livebirths, nationally and by richest and poorest wealth quintiles, by maternal mortality, stillbirth, and neonatal mortality transition phase Data from national surveys, 2000–20.

The absolute gaps in institutional births coverage between the poorest and richest wealth quintiles were largest in the early phases of the transition, reduced marginally between phases 1 to 3, and rapidly between phases 3 and 4 (from a difference of 50 percentage points to 7 percentage points; figure 5A). The pattern of inequality by household wealth changed from top inequality in phase 1 and 2, in which households in the richest wealth quintile had higher coverage than all other quintiles, to bottom inequality in phase 3 and 4, in which the poorest wealth quintile had considerably lower coverage than all other quintiles (appendix p 28).

Caesarean section rates among the poorest wealth quintiles were lower than 1% in phase 1, and still lower than 2% in phase 2. A major increase in caesarean rates occurred from phase 3 to phase 4 (from 4% to 15%; figure 5B). Among the richest quintile, the increase in caesarean section rates took place earlier from phase 2 to phase 3 (from 10% to 28%).

## Discussion

The usefulness of a transition model depends on whether it is possible to identify meaningful phases in the transition, and whether those phases have a set of characteristics that help understand past changes, current situations, and facilitate planning of future strategies. We showed that it is possible to develop a combined transition model for maternal and neonatal mortality and stillbirths. The big picture of the transition is intuitive and aligned with other transition theories.^31^, ^32^, ^33^ As mortality declined, reductions occurred in the relative share of infectious diseases and peripartum complications as a cause of death, and in adolescent and total fertility, and health-system strength and service coverage improved, overall and among the poorest wealth quintiles. Phase-specific characteristics were independent of time given that all patterns were remarkably similar in 2000 and 2020.

Deliveries in smaller health facilities had a major role in the initial transition phases, but from phase 3, hospital deliveries became predominant. In phase 4 and phase 5, the majority (but not all) of women and babies received services at higher-level facilities, and elective caesarean sections could be a challenge.^36^

A transition model allows for the identification of outlying countries. Some countries, such as Pakistan and Turkmenistan in 2020, had atypical combinations of maternal and stillbirth plus neonatal mortality, which should prompt further investigation of data quality and, if the data are considered valid, the reasons for the aberrant mortality patterns. This approach can be taken further to assess whether the mortality characteristics of a country (cause-of-death patterns, fertility, health system, coverage, inequalities, and socioeconomic development) are typical (within the IQR) for a particular phase. We have provided these median and quartile values obtained from our multicountry analyses (appendix pp 29–33). There are three applications for this approach. First, this approach allows benchmarking of the current situation of a country against common patterns on the basis of the experience of other countries at the same transition phase. Second, this approach provides a tool to assess potential drivers of progress by comparing country indicators with a previous phase during the preceding decades. Third, the transition model informs strategic planning by comparing current indicators with common patterns in subsequent transition phases.

It is beyond the scope of this Article to discuss the implications of a complex mortality transition, but one example stands out. The shift from predominance of home deliveries to lower-level health facilities and then to hospitals (which are more likely to be capable of providing comprehensive emergency obstetric and newborn care) is a central element of the transition. Almost all countries that transited from phase 1 to phase 2 and early phase 3 with major increases in coverage of antenatal and institutional birth services in lower-level health facilities need further dialogue on delivery strategies to move to subsequent phases and achieve global and national mortality targets.^37^, ^38^, ^39^

There are several limitations of the model. We used the UN estimates of mortality to classify countries. These estimates have large uncertainty intervals, especially maternal mortality, and we showed how the use of lower and upper mortality bounds leads to, on average, half a phase difference in the classification of countries. The use of common covariates in the global estimation models for maternal mortality and stillbirths (eg, using neonatal mortality as a covariate for estimates of stillbirth rates) also affects correlations between these outcomes. The strong associations between the mortality indicators in empirical studies is, however, reassuring. Given the mortality data limitations, the transition model might also be used as a tool to check data quality. Inconsistent mortality estimates should prompt further data quality considerations. Major outlying observations related to the components of the model, such as fertility or coverage of interventions, should also lead to queries about the mortality data used to classify a country according to transition phase.

In the absence of reliable death-registration systems, most mortality estimates for low-income and middle-income countries are primarily based on household surveys with sibling survival histories for maternal mortality, and birth and reproductive histories for stillbirth and neonatal mortality. Data availability and quality is a major issue, especially for stillbirths. Stillbirths are often heavily underreported in surveys, and misclassification of stillbirths and neonatal deaths is also a concern.^40^, ^41^ Neonatal death reporting is generally more complete, although omission and misclassification is a problem in some surveys.^42^

The use of neonatal mortality without stillbirths (eg, if a country analysis is based on empirical mortality data) is a viable alternative, using the same five transition phases, with neonatal mortality thresholds of 45, 30, 15 and five phase transitions per 1000 livebirths. Cause-of-death information was still poor in most low-income and middle-income countries for maternal deaths, stillbirths, and neonatal deaths.

The thresholds between phases are, to some extent, arbitrary. We used historical mortality data, thresholds from previous publications on maternal and neonatal mortality (such as the correspondence of the widespread adoption of neonatal intensive care with neonatal mortality rates of 15 per 1000 livebirths or less),^43^ and the ratio of maternal to stillbirth plus neonatal mortality to define the thresholds for the phase transitions. The phases should be interpreted as indicative, to help assess country situation, progress, and future strategies. Furthermore, the transition model should not be interpreted as a unidirectional uniform pathway towards mortality reduction. Heterogeneity in pathways, countertransitions, and variation in pace and drivers of progress are common features of all transition models.^44^ Our model intends to help recognise such developments and support strategy debates for appropriate action.

In 2020, most low-income and lower-middle-income countries had progressed to phase 2 and early phase 3 of the maternal, stillbirth, and neonatal mortality transition. The global 2030 mortality targets of the Sustainable Development Goals lie within phase 4, which is still a long way off for many countries, even if the pace of decline remains as fast as during the past two decades. The transition model suggests that moving into phase 4 and beyond will, in most settings, be accompanied by a set of concomitant changes in fertility and abortion policies, health workforce and financing, intervention coverage, hospital delivery and inequalities, and general socioeconomic progress.

## Data sharing

All data used for this study are available in the public domain. Details of all data sets are provided in the appendix (pp 1–2).

## Declaration of interests

We declare no competing interests.
