# Supplementary material for: Maternal mortality, stillbirths, and neonatal mortality: a transition model based on analyses of 151 countries
Source: Lancet Glob Health. 2023 Jun 20;11(7):e1024–31. doi: 10.1016/S2214-109X(23)00195-X (PMC10299966; doi:10.1016/S2214-109X(23)00195-X)
Supplement: Supplementary appendix [file mmc1.pdf]

THE LANCET
Global Health

Supplementary appendix

This appendix formed part of the original submission and has been peer reviewed. We post it as supplied by the authors.

Supplement to: Boerma T, Campbell OMR, Amouzou A, et al. Maternal mortality, stillbirths, and neonatal mortality: a transition model based on analyses of 151 countries. *Lancet Glob Health* 2023; **11:** e1024–31.

Supplementary materials

Maternal mortality, stillbirths, and neonatal mortality:

a transition model based on analyses of 151 countries

Annex	Title	Page
A	Data sources and definitions	1
B	Association between stillbirth and neonatal mortality rates and maternal mortality ratios	3
C	Mortality transition	8
D	Causes of death	12
E	Fertility and abortion policies	18
F	Health system changes	20
G	Service delivery coverage	23
H	Inequalities	24
I	Summary table and reference values for assessment tool	26
	References	30

A: Data sources and definitions

Mortality

Maternal mortality ratios were extracted from the WHO database for 2000 to 2020. https://www.who.int/publications/i/item/9789240068759 (accessed 23 February 2023). Details of the methods used by the UN are provided in World Health Organization.[1] The maternal mortality ratio (MMR) is defined as: the number of maternal deaths in a population per 100,000 live births during one calendar year.

Stillbirth rates and neonatal mortality rates were extracted from the UN Interagency Group for Child Mortality estimation (IGME) database (https://childmortality.org/) (accessed 12 December 2022). The following definitions are used (https://childmortality.org/methods).[2]

- stillbirth rate: the number of babies born with no sign at life at 28 weeks or more of gestation per 1,000 births, each year
- neonatal mortality rate: the number of deaths of infants under 28 days of age per 1,000 live births, each year

We computed defined stillbirth + neonatal mortality per 1,000 births as: stillbirth rate per 1,000 births + neonatal mortality rate per 1,000 live births/(1+stillbirth rate/1000)

Country population

Data were obtained from the UN Population Division database. United Nations, Department of Economic and Social Affairs, Population Division (2022). World Population Prospects 2022, Online Edition. (https://population.un.org/wpp/Download/Standard/Population, accessed February 24 2023). We excluded countries with populations of less than 1 million in 2020.

Socioeconomic indicators

Gross National Income (Atlas method, current US$) was extracted from: https://data.worldbank.org/indicator/NY.GNP.PCAP.CD (accessed 24 February 2023)

School enrollment, secondary (% gross) from : https://data.worldbank.org/indicator/SE.SEC.ENRR (accessed 24 February 2023) Gross enrollment ratio is the ratio of total enrollment, regardless of age, to the population of the age group that officially corresponds to the level of education shown. Secondary education completes the

provision of basic education that began at the primary level and aims at laying the foundations for lifelong learning and human development, by offering more subject- or skill-oriented instruction using more specialized teachers.

Causes of death - neonatal

Details of the cause of death analysis are presented in Annex D. The causes of neonatal death were obtained from the WHO Global Health Observatory. https://www.who.int/data/gho/data/indicators/indicator-details/GHO/distribution-of-causes-of-death-among-children-aged-5-years-(-) (accessed September 1 2022)

Fertility

Total fertility rates and age-specific fertility rates (15-19 years) were extracted from:
United Nations, Department of Economic and Social Affairs, Population Division (2022). World Population Prospects 2022, Online Edition.
File GEN/01/REV1: Demographic indicators by region, subregion and country, annually for 1950-2100
https://population.un.org/wpp/Download/Standard/MostUsed/

Abortion policies

Data on abortion policies and laws were obtained from the WHO database: Global Abortion Policies database. https://abortion-policies.srhr.org/ (accessed 23 Feb 2023). We developed a simple score based on the legal grounds for abortions – the higher the score, the more permissive abortion policy in the country is. Data were available for 144 countries in 2001 and 148 countries in 2021. Countries which did not permit abortion in any circumstances were scored as 1; those that permitted abortion to save the life of the mother, scored 2; health grounds were 3; broad social grounds were 4 and on demand were 5. Mean scores by phase were calculated as a percentage out of a score of 5. Further details are provided in Annex E.

Health financing

Data were extracted from the WHO Global Health Expenditure database: https://apps.who.int/nha/database/Select/Indicators/en (accessed 25 Feb 2023). No data were available for DPRK, Somalia and the State of Palestine, 148 countries remained for our analyses. No data for 2020 were available from Albania, Saudi Arabia, Libya, Yemen and Syria. Afghanistan, Iraq, South Sudan and Zimbabwe had no data for 2000.

Health workforce

Global Health Workforce Statistics, The National health Workforce Accounts database, World Health Organization, Geneva. https://www.who.int/data/gho/data/themes/topics/health-workforce (accessed 28 Feb 2023) . Because there are a substantive number of missing annual values, we used period rates as there were many years with missing data and present those as 2002 (2000-2004) and 2018 (2016-2020), matched to the transition phase in those years.

Coverage and inequalities: household surveys

For service coverage indicators, including inequalities, and neonatal mortality rates by place of birth, we analyzed data from all national Demographic and Health Surveys (DHS) and Multiple Indicator Cluster Surveys (MICS) conducted during 2000-2020. The analyses were done by the International Center for Equity in Health at the Federal University of Pelotas, Brazil. https://equidade.org/ Of the 326 household surveys, 38 were conducted when the country was in transition phase I, 126 in phase II, 71 in phase III, 76 in phase IV and 15 in phase V. The surveys were conducted in 99 low- and middle-income countries.

Historical data and prospective studies

We identified publications with historical data for the period from 1920 with consistent estimates on maternal mortality, stillbirth rates and neonatal mortality rates. We also reviewed times series from Sri Lanka and Malaysia. The prospective studies of the outcome of pregnancy were also selected based on the availability of data on all three indicators. See annex B for references.

Annex B: Associations between stillbirth and neonatal mortality rates, and maternal mortality ratios

Stillbirth and neonatal mortality rates were highly correlated. In historical data (1930-1965) for six populations,[3] [4] [5] [6] the correlation coefficient between stillbirth and neonatal mortality was 0.82 (r^2=0.68) and the median ratio stillbirth to neonatal death was 1.05 (IQR: 0.88-1.18) (Table B1 and Figure B1). There is considerable variation between the six populations which may be due to different perinatal death registration practices with variable extents of misclassification of stillbirths or neonatal deaths.

Figure B1 Relationship of neonatal mortality rate (per 1000 births) to stillbirth rate (per 1000 births), historical data for USA whites, USA non-whites, England and Wales, Norway, Netherlands, Canada, 1930-1965

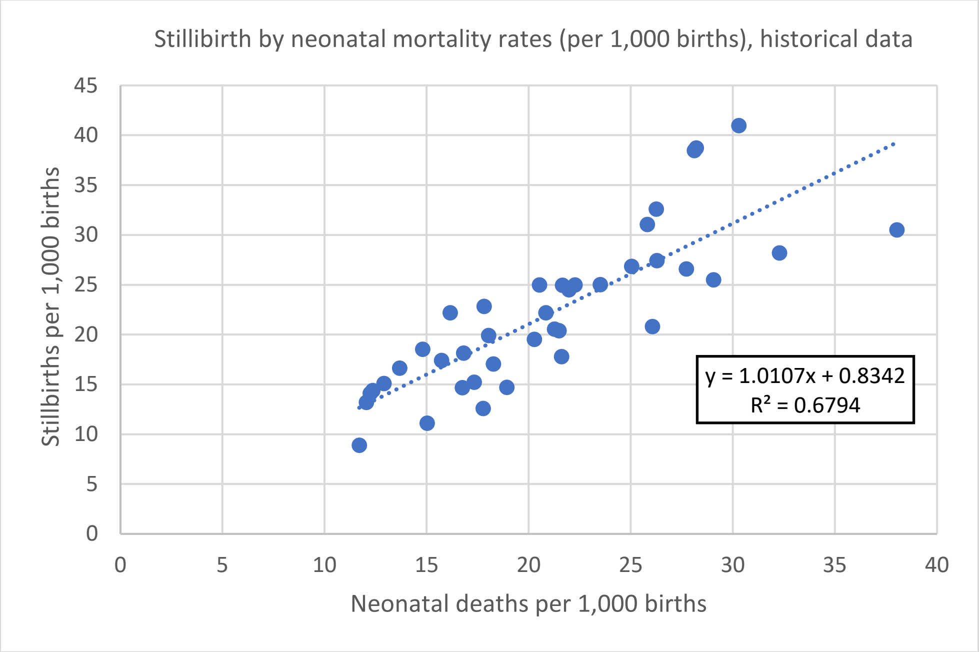

A weak correlation (r=0.29) was observed between the ratio of stillbirth to neonatal mortality rates and the rates of stillbirth and neonatal mortality. The ratio declined as levels of mortality decreases but with considerable variation (Figure B2). In 20 prospective studies on pregnancy outcomes in low- and middle-income countries, conducted since 2010, the median stillbirth to neonatal death ratio was 1.1 (IQR: 0.9-1.2). (Table B2).[7] [8] [9] [10]

The UN-IGME estimates of stillbirth and neonatal mortality rates for 2000 and 2020 are also strongly correlated (r=0.95 in both years) (Figure B3). Among countries with a stillbirth+neonatal mortality rate of 20 per 1,000 births or higher, the median stillbirth to neonatal death ratios were 0.72 (IQR: 0.65-0.81) and 0.80 (IQR: 0.72-0.91) in 2000 and 2020, respectively. In lower mortality settings (below 20), the median ratios were higher (1.0 in 2000 and 1.1 in 2020) (Figure B4). An important caveat is that the UN estimation model for stillbirths uses covariates including neonatal mortality, as well as GNI per capita, female schooling, low birthweight, antenatal care and C-section rate. [11]

Table B1 . Maternal mortality ratio (MMR), stillbirth rates (SBR), neonatal mortality rates (per 1,000 live births and per 1,000 births) in historical time series, with ratios of MMR to NMR and of MMR to stillbirth + neonatal mortality rates, selected countries

	Year	MMR	Stillbirth rate	NMR (live births)	NMRb (births)	SBR +NMR (births)	Ratio SB/NMRb	Ratio NMR to MMR	Ratio SB+NMRb to MMR
Canada	1930-34	524	31	39	38	69	0.80	7	13
	1935-39	480	28	33	32	60	0.87	7	13
	1940-44	328	26	30	29	55	0.88	9	17
	1945-49	174	21	27	26	47	0.80	15	27
	1950-54	92	18	22	22	39	0.82	24	43
	1955-59	60	15	19	19	34	0.78	32	56
	1960-64	40	13	18	18	30	0.71	45	76
	1965-69	28	11	15	15	26	0.74	54	93
	1970-74	16	9	12	12	21	0.76	74	129
England & Wales	1930-34	449	41	32	30	71	1.35	7	16
	1935-39	357	38	29	28	67	1.37	8	19
	1940-44	271	33	27	26	59	1.24	10	22
	1945-49	139	25	22	22	47	1.15	16	34
	1950-54	74	23	18	18	41	1.28	25	55
	1955-59	49	22	17	16	38	1.37	34	79
	1960-64	34	19	15	15	33	1.25	44	97
Netherlands	1920-24	238	27	28	28	54	0.96	12	23
	1925-29	315	25	24	24	49	1.06	8	15
	1930-34	318	25	23	22	47	1.12	7	15
	1935-39	273	25	21	21	46	1.22	8	17
	1940-44	209	21	22	21	42	0.97	10	20
	1945-49	143	20	21	20	40	0.96	14	28
	1950-54	83	18	17	17	35	1.08	21	42
	1955-59	56	17	14	14	30	1.22	25	54
	1960-64	35	14	13	12	27	1.16	35	76
Norway	1931-35		25	23	22	46	1.12	-	-
	1936-40		22	21	21	43	1.07	-	-
	1941-45		20	18	18	38	1.10	-	-
	1946-50		17	16	16	33	1.11	-	-
	1951-55		15	13	13	28	1.17	-	-
	1956-60		14	12	12	26	1.15	-	-
	1961-62		13	12	12	25	1.10	-	-
USA, whites	1945-49	141	20	22	21	42	0.95	16	30
	1950-54	49	17	19	18	35	0.93	38	72
	1955-59	28	15	18	17	33	0.88	62	115
	1960-62	25	15	17	17	31	0.88	69	127
USA, non-whites	1945-49	337	39	29	28	67	1.37	9	20
	1950-54	184	31	27	26	57	1.20	14	31
	1955-59	113	27	27	26	54	1.04	24	48
	1960-62	98	27	26	25	52	1.07	26	53

Figure B2 Ratio of stillbirth to neonatal deaths by level of stillbirth + neonatal mortality per 1,000 births, historical data for USA whites, USA non-whites, England and Wales, Norway, Netherlands, Canada, 1930-1965

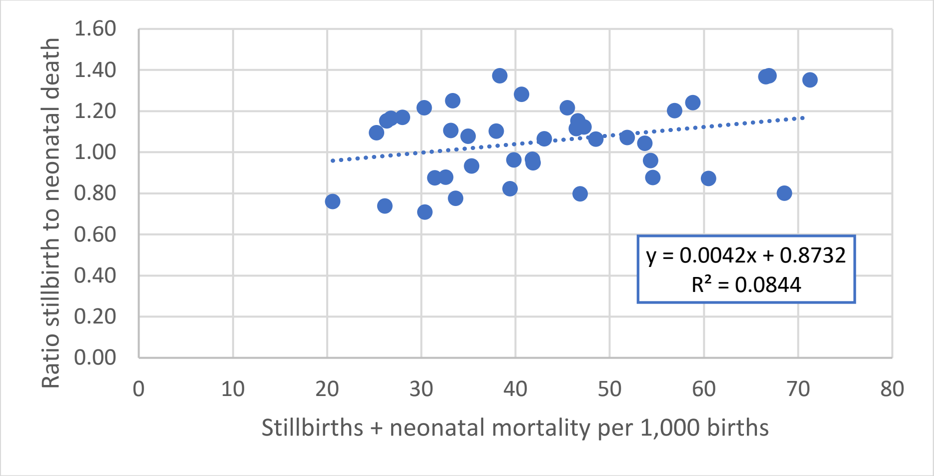

Table B2: Maternal mortality ratio (MMR), stillbirth (SBR), neonatal mortality (NMR) rates and ratios in prospective studies of pregnancy outcomes

Study	Period	Maternal mortality per 100,000 live births	Stillbirths per 1000 births	Neonatal mortality per 1000 livebirths	Stillbirth and neonatal mortality per 1000 births	Ratio SBR / NMR	Ratio SBR +NMR to maternal mortality
**Global Network Studies**							
Argentina, Corrientes	2010-12	69	14.1	10.1	24.1	1.4	35
Guatemala, Chimaltenango	2010-12	109	20.0	21.6	41.2	0.9	38
India, Belgaum	2010-12	139	25.7	23.4	48.5	1.1	35
India, Nagpur	2010-12	155	27.9	22.4	49.7	1.3	32
Kenya, Eldoret	2010-12	136	21.2	15.5	36.4	1.4	27
Pakistan, Thatta	2010-12	316	56.1	50.2	103.6	1.2	33
Zambia, Lusaka	2010-12	144	21.0	18.9	39.5	1.1	27
**AMANHI studies**							
Bangladesh, Sylhet	2012-16	456	39.0	37.8	75.4	1.1	17
India, Haryana	2012-16	191	22.3	40.0	61.4	0.6	32
India, Uttar Pradesh	2012-16	399	37.6	41.7	77.8	0.9	19
Pakistan, Matiari	2012-16	259	42.8	46.9	87.8	1.0	34
Pakistan, Karachi	2012-16	460	37.8	50.1	86.1	0.8	19
DR Congo, N + S Ubangi	2012-16	1188	24.6	28.2	52.1	0.9	4
Ghana, Brong Ahofo	2012-16	326	26.9	29.1	55.2	0.9	17
Kenya, Western	2012-16	94	7.5	12.8	20.2	0.6	21
Tanzania, Ifakara	2012-16	406	14.9	27.2	41.7	0.6	10
Tanzania, Pemba	2012-16	350	25.7	16.0	41.3	1.6	12
Zambia, Southern	2012-16	-	17.3	14.5	31.6	1.2	-
**Other studies**							
Pakistan, Tehsil/Havelian	2015/16	247	40	32	70.8	1.3	29
Malawi, Central Region	2005	377	32.2	28.6	59.9	1.2	16

Figure B3: Stillbirths per 1,000 births by neonatal mortality per 1,000 births, UN-IGME estimates, 2000 and 2020

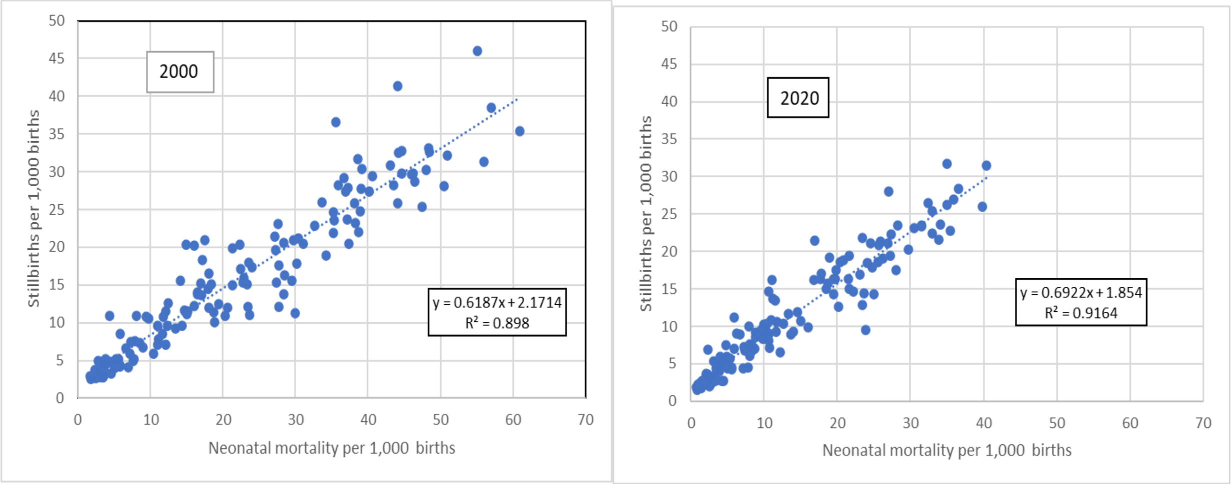

Figure B4: Ratio of stillbirth (SBR) to neonatal mortality rates (NMR-b, per 1,000 births) by level of stillbirths + neonatal mortality per 1,000 births, UN estimates 2000 and 2020

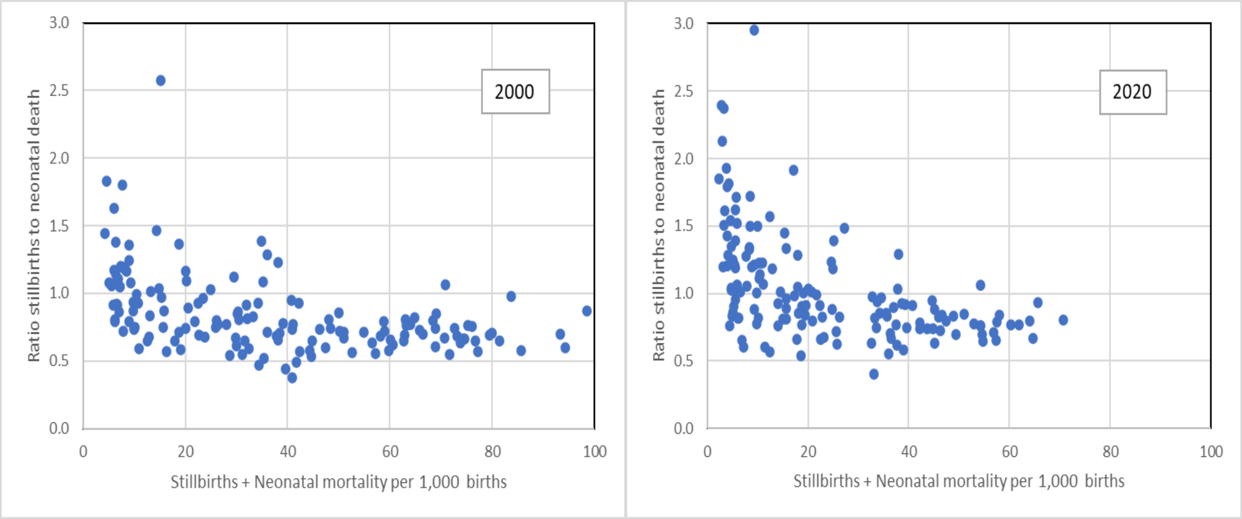

Estimates of maternal and stillbirths + neonatal mortality were also closely correlated in the different datasets, but ratios change as mortality levels decline in most settings. In five historical populations (no comparable maternal mortality data were obtained for Norway), the ratio of stillbirths + neonatal deaths to maternal deaths increased from 10-20 to levels approximating 100 (but with considerable variation) during 1930-1965 and later, reflecting a faster rate of decline of maternal mortality than stillbirths and neonatal mortality rates (Table B1 and Figure B5).

The ratio of neonatal to maternal mortality shows a similar pattern (Table B1 and Figure B6). Here, we added data from Malaysia and Sri Lanka for the period 1949-1990 (which had no stillbirth data from the same time period). The difference between these countries compared to each other and to the five other historical populations was large, with Malaysia having extremely high ratios of neonatal to maternal mortality for its mortality level in the early years, and Sri Lanka having extremely low ratios. The declining trend as mortality levels fall was evident in all countries, and some convergence of the ratios is occurring.

In 19 prospective studies of pregnancy outcomes in contemporaneous low- and middle-income country settings (the DR Congo site was excluded as a major outlier with extremely high maternal mortality compared to stillbirth

and neonatal mortality rates), the median ratio stillbirth + neonatal deaths to maternal deaths is 27 (IQR: 17-33), and the ratios did not vary systematically by mortality levels (Table A.2).[12] [13] For neonatal to maternal mortality the median ratio was 13 (IQR 9-15).

UN estimates of stillbirths + neonatal mortality and maternal mortality were also highly correlated and show a similar association with of the ratio with mortality levels. The ratio increased gradually until low levels of stillbirth + neonatal mortality are reached, when further major increases were observed. The ratio is also useful to identify outliers (Figure B7).

Figure B5: Historic trends in the ratio of stillbirth+neonatal to maternal deaths, by stillbirth + neonatal mortality per 1,000 births in five European and North American populations, 1930-1965.

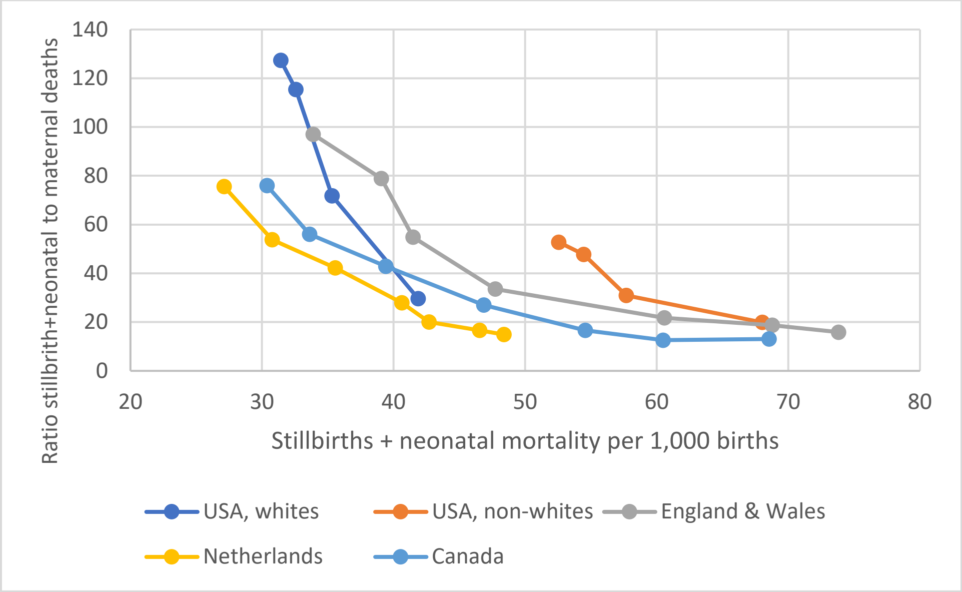

Figure B6: Trends in the ratio of neonatal to maternal deaths by neonatal mortality per 1,000 births in European and North American populations, 1930-1965, and in Malaysia and Sri Lanka, 1949-1990.

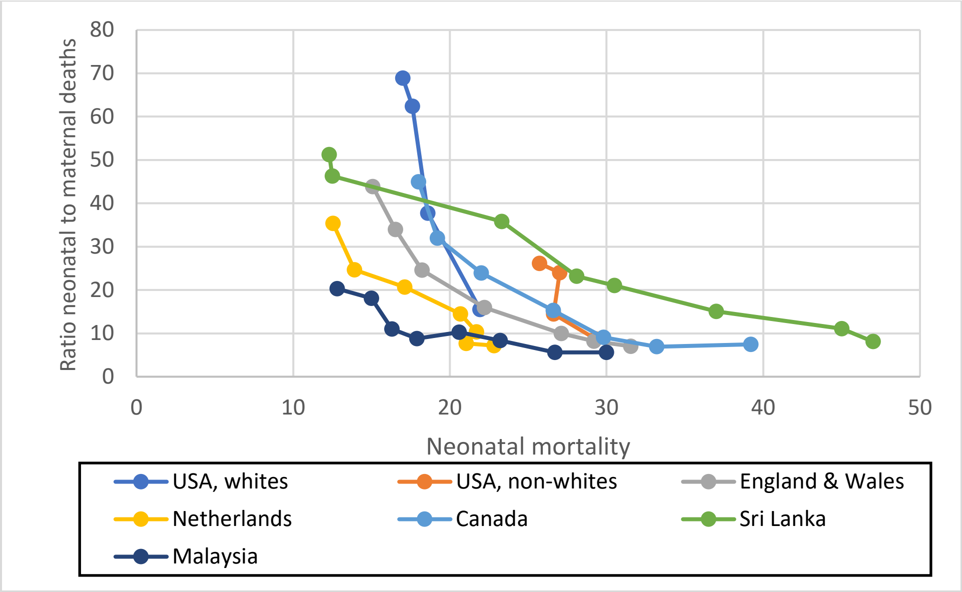

Figure B7: Ratio of stillbirths + neonatal deaths to maternal deaths by level of stillbirths + neonatal mortality per 1,000 births, UN (IGME and MMEIG) estimates, 151 countries, 2000 and 2020.

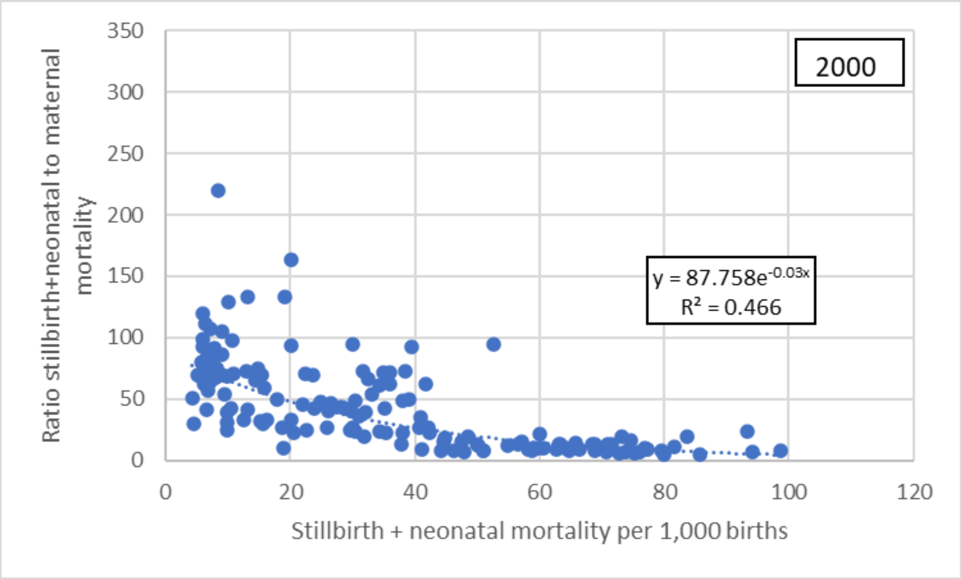

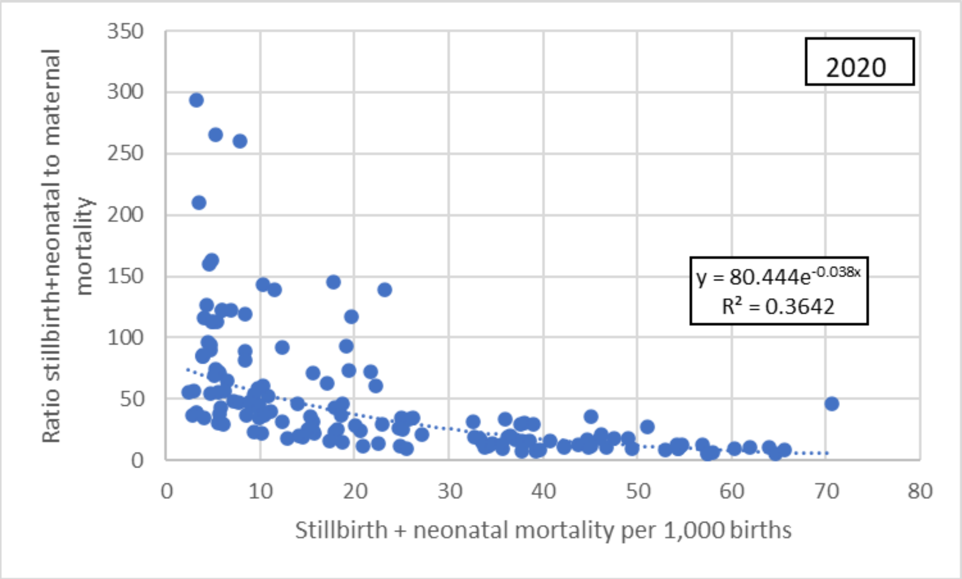

*For 2020 the graph does not include Turkmenistan which had an improbable ratio of 644 duei to extremely low maternal mortality in comparison to stillbirth + neonatal mortality estimates.

Annex C: Mortality transition

The ratio of maternal deaths to 100 perinatal deaths based on the UN estimates for 2000 and 2017 for countries with a population exceeding 1 million in 2000 is shown in Table C1. The median ratio increased from under 10 in Phase I (high mortality countries) to almost 100 in Phase V (low mortality) of the transition.

Table C1: Number of countries by transition phase, with mean, median, Q1 and Q3 values of the ratio of stillbirth + neonatal mortality to maternal mortality, 149 countries, based on UN estimates

Phase	N countries	Mean	Median	Q1	Q3
2000					
I	21	8.7	7.6	6.5	8.5
II	29	12.1	12.5	9.7	13.6
III	34	45.6	37.5	22.8	61.9
IV	32	51.8	42.0	32.4	62.6
V	35	83.7	74.9	68.3	98.2
Total	151	44.2	32.7	12.9	69.0
2020					
I	5	6.4	5.5	5.4	6.9
II	23	11.9	10.3	9.4	12.0
III	32	39.0	17.6	14.9	24.6
IV	42	44.8	34.6	25.4	46.4
V	49	96.0	84.3	55.1	118.7
Total	151	53.9	33.1	15.7	69.3

Table C2 presents the distribution of countries by subregion of the world and phase of the mortality transition. By 2017, the majority of countries in sub-Saharan Africa are in phases I and II, in South Asia in II and III, in East Asia and Pacific in III to V, in Latin America & Caribbean and Middle East and North Africa in phase IV, in Eastern Europe and Central Asia in phases IV and V and in Western Europe and North America in phase V.

Table C2: Number of countries by mortality transition phase and region in 2000 and 2017.

	2000						2020					
	I	II	III	IV	V	Total	I	II	III	IV	V	Total
Eastern and Southern Africa.	8	11	2	1	0	22	1	8	12	1	0	22
West and Central Africa	10	10	1	0	0	21	4	12	5	0	0	21
South Asia	3	2	0	1	0	6	0	2	3	1	0	6
East Asia and Pacific	0	4	5	3	5	17	0	0	7	5	5	17
Middle East and North Africa	0	1	7	6	2	16	0	0	1	11	4	16
Latin America and Caribbean	0	1	10	11	0	22	0	1	3	16	2	22
Eastern Europe and Central Asia	0	0	9	8	3	20	0	0	1	7	12	20
Western Europe and North America	0	0	0	2	25	27	0	0	0	1	26	27
Total	21	29	34	32	35	151	5	23	32	42	49	151

Table C.3 presents the transition phase and mortality estimates for all 149 countries by region, for the years 2000 and 2017, obtained from the Maternal Mortality Estimation Inter-Agency Group and the Interagency Group on

Child Mortality estimation. The stillbirth and neonatal mortality rate was computed from the stillbirth rates and neonatal mortality rates, with births as the common denominator.

Table C3: Country mortality transition phase and UN mortality estimates, grouped by regions, 2000 and 2017 (MMR: maternal mortality ratio per 100,000 live births, stillbirth and neonatal mortality rate, per 1,000 births (SBNMR), SB: stillbirth rate, per 1,000 births; NMR: neonatal mortality rate, per 1,000 live births)

	2000					2020				
country	phase	MMR	SBNMR	SBR	NMR	phase	MMR	SBR	NMR	SBNMR
Eastern and Southern Africa										
Angola	I	860	77	28	50	III	222	19	27	46
Botswana	III	182	19	11	8	III	186	15	19	33
Burundi	I	874	65	29	37	II	494	19	21	39
Eritrea	I	735	48	21	27	II	322	16	18	34
Eswatini	II	588	44	16	29	III	240	14	24	38
Ethiopia	I	953	80	33	48	III	267	21	27	47
Kenya	II	564	46	20	27	II	530	19	19	38
Lesotho	II	545	71	37	36	II	566	27	36	62
Madagascar	II	658	51	20	31	II	392	19	24	42
Malawi	II	573	60	22	39	II	381	16	20	36
Mauritius	IV	52	25	13	12	IV	84	10	10	21
Mozambique	II	532	72	25	47	III	127	17	28	45
Namibia	II	450	41	18	24	III	215	17	20	37
Rwanda	I	1007	71	28	44	III	259	17	18	35
Somalia	I	1097	76	33	45	II	621	28	37	64
South Africa	III	173	38	21	17	III	127	16	11	27
South Sudan	I	1687	86	31	56	I	1223	26	40	65
Sudan	II	642	64	28	37	III	270	22	27	49
Uganda	II	461	55	23	33	III	284	16	19	36
United Republic of Tanzania	I	760	59	26	34	III	238	19	20	39
Zambia	II	419	56	22	35	III	135	14	25	39
Zimbabwe	II	388	50	23	28	II	357	19	26	44
West and Central Africa										
Benin	II	469	66	28	39	II	523	20	30	49
Burkina Faso	II	506	69	29	41	III	264	21	26	46
Cameroon	II	651	58	24	35	II	438	19	26	45
Central African Republic	I	1315	73	31	43	I	835	26	32	58
Chad	I	1366	75	32	44	I	1063	25	33	58
Congo	II	660	51	21	30	III	282	16	19	34
Cote d'Ivoire	II	473	75	30	46	II	480	22	33	55
Democratic Republic of the Congo	II	668	69	32	39	II	547	28	27	54
Gabon	III	249	45	18	28	III	227	14	19	34
Gambia	I	778	66	27	40	II	458	21	26	47
Ghana	II	499	63	28	36	III	263	22	23	45
Guinea	I	971	74	30	46	II	553	23	32	54
Guinea-Bissau	I	1300	99	46	55	I	725	32	35	66
Liberia	I	777	77	30	48	II	652	23	30	53
Mali	I	742	82	32	51	II	440	24	34	57

Mauritania	II	684	63	25	39	II	464	17	23	40
Niger	I	867	69	26	44	II	441	22	34	55
Nigeria	I	1148	74	29	46	I	1047	23	35	57
Senegal	II	638	63	26	38	III	261	19	22	41
Sierra Leone	I	1682	80	33	48	II	443	23	32	54
Togo	II	479	63	27	37	II	399	21	25	45
Latin America and Caribbean										
Argentina	IV	72	19	8	11	IV	45	5	6	10
Bolivia (Plurinational State of)	III	284	45	16	29	III	161	9	14	23
Brazil	IV	68	29	10	19	IV	72	7	9	16
Chile	IV	33	10	4	6	V	15	3	4	7
Colombia	IV	93	23	9	14	IV	75	7	7	14
Costa Rica	IV	39	13	5	8	IV	22	4	6	10
Cuba	IV	47	15	11	4	IV	39	7	2	9
Dominican Republic	III	79	38	15	23	III	107	13	23	36
Ecuador	III	120	30	16	14	IV	66	9	7	16
El Salvador	III	49	35	20	15	IV	43	9	6	15
Guatemala	III	152	41	20	21	IV	96	14	11	25
Haiti	II	400	50	21	30	II	350	18	25	42
Honduras	III	82	32	14	18	IV	72	8	10	18
Jamaica	III	83	35	18	17	IV	99	15	11	25
Mexico	IV	57	24	10	14	IV	59	7	8	15
Nicaragua	III	169	32	15	17	IV	78	10	8	18
Panama	IV	66	26	12	15	IV	50	8	8	16
Paraguay	III	148	34	17	18	IV	71	10	10	20
Peru	III	113	30	14	16	IV	69	7	7	14
Trinidad and Tobago	IV	74	30	12	18	IV	27	9	11	19
Uruguay	IV	27	16	7	8	V	19	5	4	9
Venezuela (Bolivarian Republic of)	IV	92	20	10	11	III	259	11	15	26
Middle East and North Africa										
Algeria	III	159	42	20	22	IV	78	10	16	26
Egypt	III	79	39	17	22	IV	17	9	10	20
Iran (Islamic Republic of)	III	44	32	12	19	IV	22	7	8	16
Iraq	III	117	41	17	24	IV	76	12	15	26
Jordan	IV	64	28	12	16	IV	41	9	9	18
Kuwait	V	10	13	7	7	V	7	5	5	10
Lebanon	IV	32	22	11	12	IV	21	6	5	11
Libya	IV	57	26	12	15	IV	72	7	6	13
Morocco	III	244	48	21	28	IV	72	14	12	25
Oman	V	20	15	8	7	V	17	6	5	10
Saudi Arabia	IV	22	20	9	12	V	16	4	3	8
State of Palestine	III	62	30	14	17	IV	20	10	10	19
Syrian Arab Republic	IV	34	24	12	12	IV	30	11	11	22
Tunisia	III	62	33	15	18	IV	37	11	12	22
United Arab Emirates	IV	22	14	8	6	V	9	5	4	8
Yemen	II	275	60	24	37	III	183	23	28	51
Eastern Europe and Central Asia										
Albania	IV	14	19	7	12	V	8	4	7	11
Armenia	III	50	36	20	16	IV	27	11	6	17

Azerbaijan	III	56	53	19	34	IV	41	9	10	19
Belarus	IV	24	10	5	5	V	1	2	1	3
Bosnia and Herzegovina	V	16	11	4	7	V	6	3	4	7
Bulgaria	IV	22	15	8	8	V	7	5	3	8
Croatia	V	11	11	5	6	V	5	3	3	6
Georgia	III	53	38	16	23	IV	28	6	5	11
Kazakhstan	III	56	34	11	24	V	13	8	5	12
Kyrgyzstan	III	87	31	11	20	IV	50	7	12	19
North Macedonia	IV	12	20	11	9	V	3	4	4	8
Republic of Moldova	III	49	32	12	21	IV	12	7	11	18
Romania	IV	50	16	6	10	V	10	3	3	6
Russian Federation	IV	52	16	7	9	V	14	4	2	6
Serbia	V	18	13	5	8	V	10	5	4	8
Tajikistan	III	68	42	14	28	IV	17	9	14	23
Turkmenistan	III	26	41	11	30	III	5	10	24	33
Türkiye	IV	32	30	11	19	V	17	4	5	9
Ukraine	IV	36	18	7	11	V	17	5	5	10
Uzbekistan	III	43	40	12	28	IV	30	6	8	14
South Asia										
Afghanistan	I	1346	94	35	61	II	620	26	35	60
Bangladesh	I	441	84	41	44	III	123	21	17	38
India	II	384	73	30	45	III	103	13	20	33
Nepal	II	504	68	30	39	III	174	16	17	33
Pakistan	I	387	93	38	57	II	154	32	40	71
Sri Lanka	IV	61	20	11	10	IV	29	6	4	10
East Asia and Pacific										
Australia	V	7	7	4	4	V	3	2	2	5
Cambodia	II	606	59	25	35	III	218	12	13	25
China	III	58	36	15	21	IV	23	5	3	9
Democratic People's Republic of Korea	III	186	42	15	27	III	107	9	9	17
Indonesia	III	299	38	15	23	III	173	9	12	21
Japan	V	9	4	3	2	V	4	2	1	2
Lao People's Democratic Republic	II	579	61	23	38	III	126	16	22	38
Malaysia	IV	40	10	5	5	IV	21	5	4	9
Mongolia	III	158	35	12	23	IV	39	4	8	12
Myanmar	II	371	57	20	37	III	179	15	22	37
New Zealand	V	11	7	4	4	V	7	3	3	5
Papua New Guinea	II	312	47	18	30	III	192	15	22	36
Philippines	III	129	30	14	17	IV	78	10	13	23
Republic of Korea	V	16	6	3	3	V	8	2	1	3
Singapore	V	15	5	3	2	V	7	2	1	3
Thailand	IV	48	22	10	12	IV	29	6	5	10
Viet Nam	IV	97	26	11	15	III	124	8	11	19
Western Europe										
Austria	V	6	6	3	3	V	5	2	2	5
Belgium	V	8	6	3	3	V	5	3	2	5
Canada	V	9	7	3	4	V	11	3	3	6
Czechia	V	8	6	3	3	V	3	3	2	4
Denmark	V	8	7	3	3	V	5	2	3	4

Estonia	IV	25	10	5	5	V	5	2	1	3
Finland	V	7	5	3	2	V	8	2	1	3
France	V	9	8	5	3	V	8	3	3	6
Germany	V	7	6	3	3	V	4	3	2	5
Greece	V	4	8	5	4	V	8	3	2	6
Hungary	V	15	10	4	6	V	15	4	2	6
Ireland	V	10	9	5	4	V	5	3	2	5
Israel	V	9	8	4	4	V	3	3	2	5
Italy	V	10	6	3	3	V	5	2	2	4
Latvia	IV	32	13	6	7	V	18	3	2	6
Lithuania	V	18	9	5	5	V	9	3	2	5
Netherlands	V	13	9	5	4	V	4	2	3	5
Norway	V	6	6	4	3	V	2	2	1	3
Poland	V	8	10	4	6	V	2	2	3	5
Portugal	V	11	7	4	3	V	12	2	2	4
Slovakia	V	9	9	4	5	V	5	3	3	6
Slovenia	V	12	7	4	3	V	5	3	1	4
Spain	V	5	6	3	3	V	3	2	2	4
Sweden	V	6	6	4	2	V	5	2	1	4
Switzerland	V	8	6	3	3	V	7	2	3	5
United Kingdom	V	11	8	4	4	V	10	3	3	6
United States	V	12	8	3	5	IV	21	3	3	6

We classified countries based on the lower and upper limits of the uncertainty ranges of the mortality estimates. We either used all lower bounds (maternal, stillbirth and neonatal mortality) or all upper bounds and compared the distribution of countries with the classification obtained when using the point estimates (Table C4). The resulting distributions are markedly different, especially for phase I and phase V.

The mean phase based on the point estimates was 3.2, based on the lower mortality bounds 3.4 and 2.9 based on the upper mortality bounds.

Table C4: Countries classified by transition mortality phase based on the lower and upper bounds of the UN mortality estimates, 151 countries, 2000 and 2020

2000	Lower (N)	Point est. (N)	Upper (N)	Lower (%)	Point est. (%)	Upper (%)
I	12	21	40	7.9	13.9	26.5
II	35	29	18	23.2	19.2	11.9
III	27	34	38	17.9	22.5	25.2
IV	37	32	25	24.5	21.2	16.6
V	40	35	30	26.5	23.2	19.9
Total	151	151	151	100.0	100.0	100.0
2020						
I	3	5	19	2.0	3.3	12.6
II	19	23	30	12.6	15.2	19.9
III	31	32	26	20.5	21.2	17.2
IV	36	42	35	23.8	27.8	23.2
V	62	49	41	41.1	32.5	27.2
	151	151	151	100.0	100.0	100.0

Annex D: Causes of death

A common cause of death structure, linked to preventive and curative interventions, is feature of an integrated transition framework. The main causes for maternal death,[14] stillbirth[15] and neonatal mortality[16] were combined into three broad groups (Table D1).

The first group of infectious diseases includes maternal puerperal sepsis and septic abortion, maternal infections such as malaria and syphilis affecting fetal survival, and neonatal sepsis, pneumonia and tetanus. The second group includes causes related to the woman's or baby's health and nutritional status, such as non-communicable diseases and their risk factors and other non-obstetric or indirect causes, prematurity and small-for-gestational age and congenital malformations. The third group of peri-partum complications, linked to the process of birth, includes obstetric complications such as hemorrhage and hypertensive disorders of pregnancy, intrapartum conditions leading to maternal, late fetal and neonatal death due to obstructed labour and birth trauma and asphyxia, placental abnormalities and cord complications, long gestation (>41 weeks) and hypothermia.

Unsafe abortions are included in group 1, since the sepsis is the main adverse fatal consequence. One could however also argue for abortion to be included in the third cause group.

Table D1: Cause-of-death groupings for an integrated maternal mortality, stillbirth and neonatal mortality transition model

Group	Cause groups	Maternal	Stillbirth	Neonatal
1	Infections	Sepsis; Malaria; HIV; Tetanus; Unsafe abortion	Sepsis; Syphilis; Malaria; HIV	Sepsis; Syphilis; Malaria; Pneumonia; Diarrhoea; Tetanus
2	Maternal / fetal / newborn health status	Malnutrition / anemia; Short stature Diabetes / NCDs	Prematurity and small for gestational age; Congenital anomalies	Prematurity and small for gestational age; Congenital anomalies
3	Peripartum causes	Delivery complications: Hemorrhage; Obstructed labour leading to infection or hemorrhage; Hypertensive disorders of pregnancy	Obstructed labour leading to encephalopathy (birth asphyxia); Placental abnormalities; Cord complications; Long gestation (>42 weeks)	Obstructed labour leading to encephalopathy (birth asphyxia); Placental abnormalities; Cord complications; Hypothermia

The epidemiological transition model posits a decline in infectious diseases and nutritional improvements, and an increase in NCDs, as all-cause mortality levels decrease. For the maternal and peri-neonatal transition this would imply a transition from group 1 to group 2. The maternal and perinatal mortality transition however is more complex as the third group, peripartum complications, plays a major role. Furthermore, it is noted that the proposed groups for the maternal and peri-neonatal mortality transition do not map fully on to the International Classification of Disease (ICD) groupings (where most causes in Group 3 would be part of Group 1 causes in the ICD). In the ICD, maternal and neonatal deaths due to conditions during birth are part of cause group 1, with infections and malnutrition.

Maternal deaths

Historical data on maternal mortality in several European countries show how the introduction of clean birthing practices and later the introduction of sulfonamides and antibiotics rapidly reduced to levels below 300 maternal deaths per 100,000 live births.[17] For instance, in England and Wales puerperal sepsis caused more than 30% of all maternal deaths during 1931-35 and by 1941-45 its contribution had decreased to about 15%, as maternal mortality halved from its mid-thirties level of about 430 per 100,000 live births (Figure D1).[18] [19] A decline in

mortality due to septic abortion contributed as well. Historical data from Sri Lanka also showed a decline from 25% to 5% in the contribution of sepsis as maternal mortality dropped from 580 to 65 per 100,000 live births infections during 1952 to 1980.[20] The share of mortality due to other main causes declined more gradually – due to interventions such as C-section, assisted deliveries, blood transfusion and uterotonics - resulting in greater prominence of conditions related to health of the woman (group 2).[21 22]

Figure D1: Long-term trends in major causes of death in England and Wales and Sri Lanka

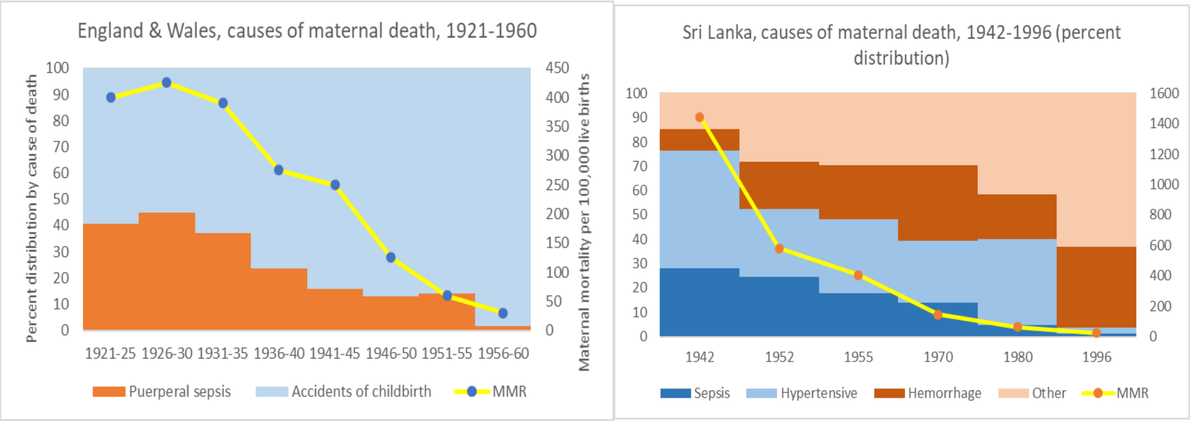

The WHO estimates of causes of maternal death, published most recently in 2014, referred to the period 2003-2009.[23] Data were only available by major region (Table 1 in their paper), showing the prominence of Group 3 with peri-partum conditions (Figure D2). We expect the Group 1 causes of infectious origin and abortion complications (often septic abortion) to be more prominent in higher mortality regions, which was indeed observed with higher proportions in developing countries than in developed countries. Obstetric causes, including hemorrhage, pregnancy-induced hypertension, embolism and other direct obstetric causes (Group 3) were more prominent in developed regions, indicating no transition in the expected direction. Indirect causes of maternal death (Group 2), which include HIV, were a little less prominent in developed countries (25%) compared to developing countries (28%) and the two highest mortality regions of South Asia and sub-Saharan Africa (29%).

Figure D2: Distribution of groups of causes of maternal death by region for 2003-2009, WHO 2014 estimates

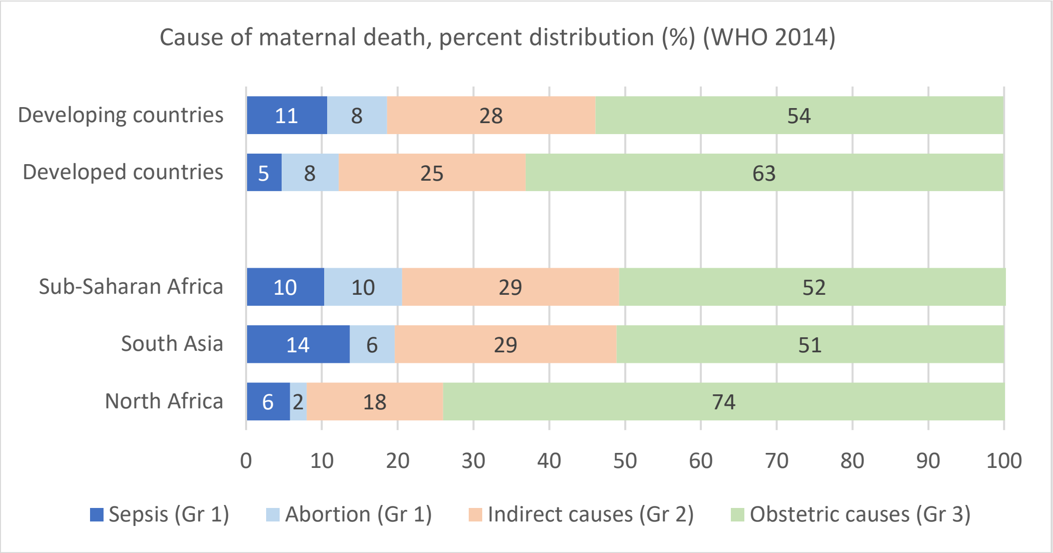

Graham and colleagues analysed the global estimates of the 2013 Global Burden of Disease study for 1990 and 2013 (Table D2).[24 25] These estimates suggest a major decline in the relative importance of infectious diseases, but not abortion, a modest change in group 3 of peri-partum / obstetric complications, and little change in group 2 of indirect causes. It was noted that the proportion association with indirect causes in the GBD estimates was unexpectedly low (12%) and not corresponding with many other studies, where it was at least two times higher. This means that the relative importance of group 2 causes is likely underestimated in the GBD.

Table D2: Distribution of maternal causes, global, based on GBD 2013, from Graham et al. 2016

	1990	2013
Group 1: infectious diseases	10	9
Group 1: Abortion complications	15	18
Group 2: indirect causes / maternal health and nutritional status	12	13
Group 3: peri-partum / obstetric conditions	61	60

The subsequent edition of the GBD (2016) that provided estimates of maternal causes showed that globally, while maternal mortality reduced by almost half during 1990-2015, the cause distribution between the main groups hardly changed. Group 1 became less prominent, primarily because of decline in abortion-related mortality from 14% to 12% of all maternal deaths. Two-thirds of all deaths remained in group 3 (obstetric causes) while indirect causes played a modest role.

Using the IHME classification of countries with low, lower middle, middle and high social development index (SDI), the emerging patterns do not particularly support a transition in causes of maternal death (Figure D3). There was no change in cause distribution at all for the low SDI countries, global or by country group according to the GBD SDI, even though the maternal mortality ratio in this group fell from 561 in 1990 to 443 in 2015. It is noted that this change in maternal mortality ratio is all within phase II of the mortality transition, but the small proportion attributed to sepsis/infections in 1990 is notable. Similarly, little change was observed in the other country groups, except the high-income countries where a major drop in abortion complications as a cause of death occurred, leading to major shift in the cause distribution.

Figure D3: Causes of maternal death, percent distribution, GBD 2016 for the years 1990 and 2015

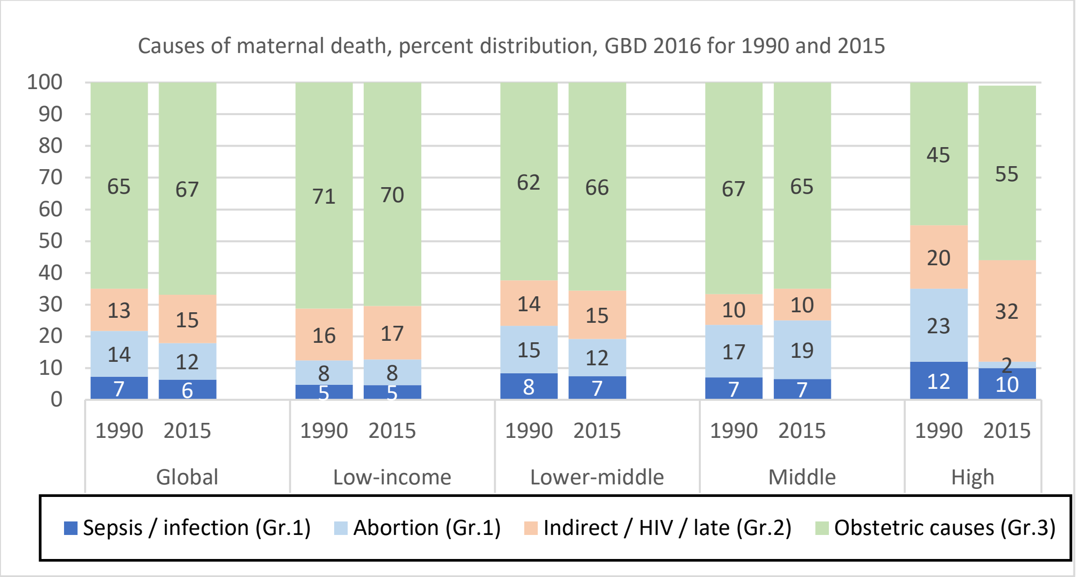

The major differences between the WHO and the GBD cause patterns is concerning. The lack of a clear association with levels may be because the simple three group classification used in the transition model hides subtle changes within the groups. A major reason however is probably the lack of reliable data on maternal mortality in especially the higher mortality countries and the large differences in cause attribution and coding practices around the world.

Neonatal deaths

We used the cause of death distributions by country from the 2021-22 update of the Maternal and Child Epidemiology and Estimation (MCEE) group. The model is described elsewhere. Data on the causes of neonatal death are scarce for higher mortality countries and the statistical models use level of neonatal / child mortality as one of the co-variates to predict cause patterns.[26]

We grouped the causes into infectious conditions (including neonatal sepsis, pneumonia, diarrhoeal diseases, neonatal tetanus, meningitis, HIV, TB), health status of the newborn (prematurity, low birth weight, congenital anomalies) and delivery complications (birth trauma, asphyxia). Other conditions which included injuries and other conditions were distributed proportionally over the three groups.

The declining proportion of deaths due to Group 1 by level of neonatal mortality is shown for the year 2000 in Figure D4, the increasing proportion due to Group 2 causes in Figure D5 and the declining proportion due to Group 3 causes in Figure D6. The trends in the major groups between 2000 and 2019 are shown in the main paper.

Figure D4: Percent of neonatal deaths due to Group 1 (infectious diseases), by neonatal mortality level, MCEE, 2000

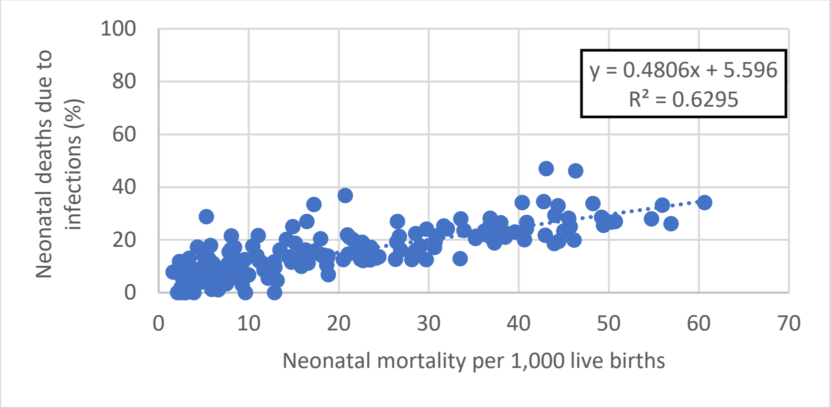

Figure D5: Percent of neonatal deaths due to Group 2 (underlying neonatal health status), by neonatal mortality level, MCEE, 2000

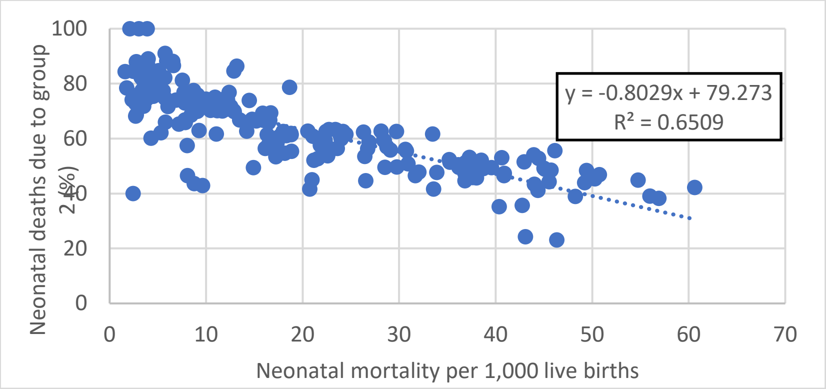

Figure D6: Percent of neonatal deaths due to Group 3 (peri-partum complications), by neonatal mortality level, MCEE, 2000

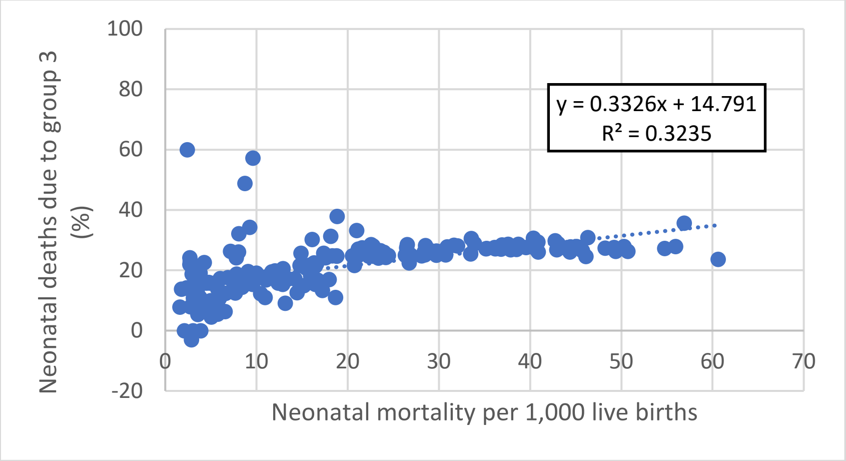

Annex E: Fertility

In most low- and middle-income countries with neonatal and fertility trend data before 1980, the neonatal mortality decline either preceded the fertility decline or ran in parallel. For instance, 59 of 62 countries with data for both indicators from 1970-74 already had ongoing neonatal mortality declines. Among these 59 countries, 45 (76%) had contemporaneous fertility declines and 14 (24%) had no fertility decline yet (Table E1).

There is a strong association between total fertility rates and phases of the transition. The country median for total fertility in 2000 was 5.9 in Phase I (IQR: 5.6-6.7), declining to 4.5 (3.9-5.5), 2.8 (2.2-3.4), 2.2 (1.6-2.7) and 1.5 (1.3-1.8) in the subsequent phases. The fertility levels by transition phase were similar in 2017. There was however considerable variability between countries within phases. In 2017, total fertility among 29 Phase II countries ranged from about 3 in Haiti, Eswatini and Lesotho, to 6 or higher in Mali, DR Congo and Niger. In 24 Phase III countries, four Asian countries had total fertility below 2.3 (Nepal, Bangladesh, India and Myanmar) while three sub-Saharan African countries (Angola, Mozambique and Zambia) had double those fertility rates. Five of 40 countries in Phase IV still had total fertility rates above 3.0 (Kyrgyzstan, Egypt, Tajikistan, State of Palestine, and Iraq), and three of the 49 countries in Phase V had total fertility rates above 2.5 (Israel, Oman and Kazakhstan).

Table E1: Number of low- and middle-income countries with neonatal mortality decline*, and timing of the fertility decline in the same time period or later onset, historic data by five-year period.

	N of countries with data	Number of countries with NMR decline	Number of countries with a contemporaneous TFR decline	Number of countries with a later TFR decline	% with NMR decline but no contemporaneous TFR decline
1960-64	29	28	16	12	42.9
1965-69	43	42	28	14	33.3
1970-74	62	59	45	14	23.7
1975-80	76	75	61	14	18.7

*Decline is defined as negative trend within the respective five-year period.

Abortion policy score

Data were available for 144 countries in 2001 and 151 countries in 2021. The summary score of abortion policies shows that countries in Phase I were most restrictive, while those in Phase V were the most permissive, with countries permitting abortion on request or on broad health and socio-economic groups (Figure D.1). Abortion policies may also be a crude proxy for the status and empowerment of women, and the extent of patriarchal norms.

Figure E1: Mean abortion policy score, 2001 and 2017, by transition phase,

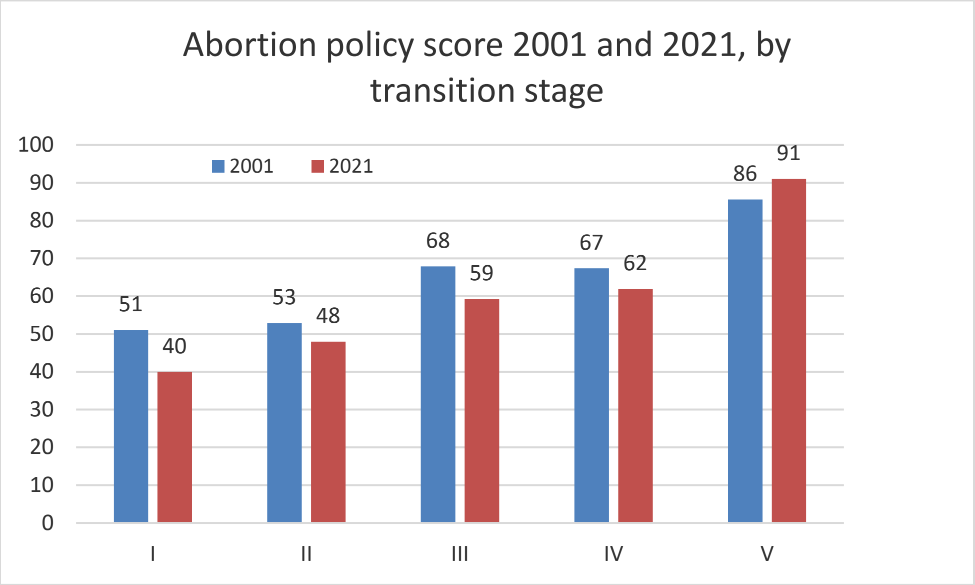

Annex F Socioeconomic status

Economic status

The GNI per capita by phase of the transition is shown in Table F1 for 2000 and 2020. Figure F1 presents the boxplot of GNI per capita on log scale (ln).

Table F1: Gross National Income per capita by mortality transition phase, 2000 and 2020

2000	N	p50	p25	p75		2020	N	p50	p10	p25	p75
I	17	280	200	400		I	4	685	460	545	1380
II	29	360	280	590		II	22	845	460	540	1420
III	33	1370	640	1770		III	28	1825	740	1090	3405
IV	31	3560	1710	4260		IV	41	4490	1740	3350	7820
V	34	21595	6840	26810		V	48	22710	6400	13905	44785
Total	144	1585	455	4875		Total	143	4490	740	1520	15300

Figure F1: Gross National Income per capita (ln scale) by mortality transition phase, 2000 (left panel) and 2020 (right panel)

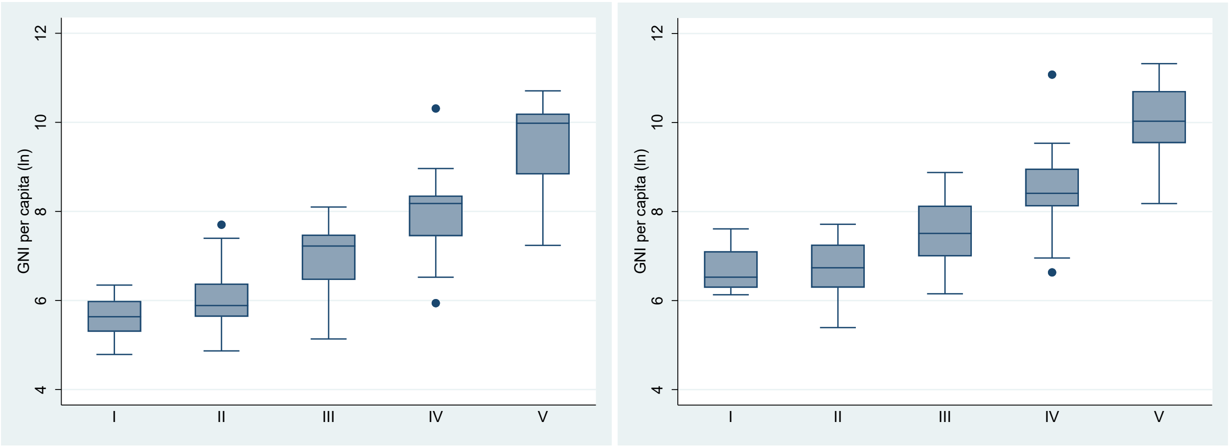

Education

The indicator selected is gross enrollment of females. It is defined as the ratio of total enrollment, regardless of age, to the population of the age group that officially corresponds to the level of education shown. Secondary education completes the provision of basic education that began at the primary level and aims at laying the foundations for lifelong learning and human development, by offering more subject- or skill-oriented instruction using more specialized teachers. Enrollment rates over 100% are possible as the indicator includes students of all ages, including students whose age is different from the official age group. Llate enrollment, early enrollment, or grade repetition, means that the total student enrollment can exceed the population of the age group that officially corresponds to the level of education – leading to ratios greater than 100 percent.

As there were many missing country years, we used data from 1999, 2000 and 2001 t o obtain a value for the year 2000, and 2019, 2020 and 2021 to obtain a value for 2020. Data were available for 122 countries in 2000 and 107 countries in 2020. A doubling of the proportion of girls with secondary school enrollment occurred from phase I to phase II and then again from phase II to phase III. This was observed in both 2000 and 2020 (Table F2 and Figure F2).

Table F1: Gross secondary enrollment in education among females by mortality transition phase, 2000 and 2020

			2000					2020	
2000	N	p50	p25	p75		N	p50	p25	p75
I	16	15.5	11.7	24.4		1	20.6	20.6	20.6
II	22	33.1	21.8	38.8		16	45.0	37.2	52.5
III	29	72.9	58.3	81.0		17	73.6	46.6	79.8
IV	23	79.8	71.1	92.4		27	98.0	89.4	102.6
V	32	100.0	93.7	107.9		46	104.8	100.9	116.9
Total	122	73.3	35.9	92.8		107	98.0	73.6	105.5

Figure F2: Gross secondary education, females, by mortality transition phase, 2000 (left panel) and 2020 (right panel)

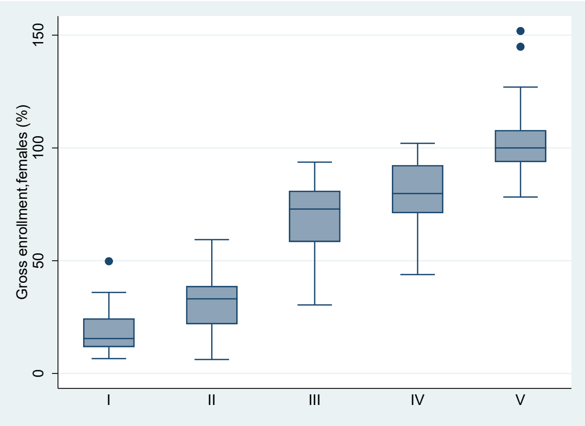

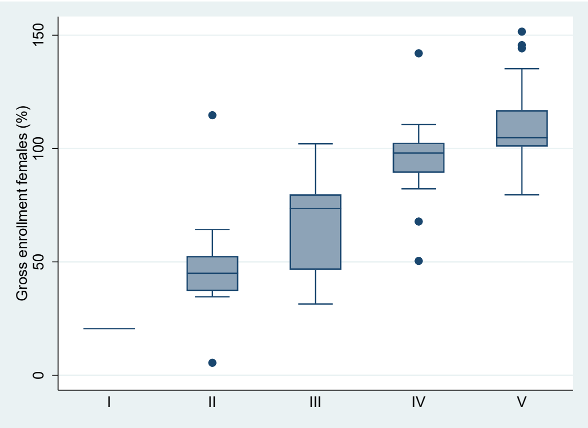

Annex G Health system changes

To assess changes in health systems, we considered phase-specific changes in total health expenditure per capita, total health expenditure as a percent of GDP, and out-of-pocket expenditure as a percent of total health expenditure, as well as density of core health professionals (physicians, nurse-midwives) and skills mix (nurse-midwives to physicians).

Health expenditure

For health financing we used data from WHO global health expenditure database to compute means for each country or seven-year period surrounding 2000 and 2020. [27] There is good consistency in the findings for the three years / time periods. Only for out of pocket spending there is considerable variation: in the earlier years higher levels were observed in the first three phases (Figures G1-G3 and Table G1).

Figure G1 Total health expenditure per capita, 2003, 2010, 2017, by transition phase

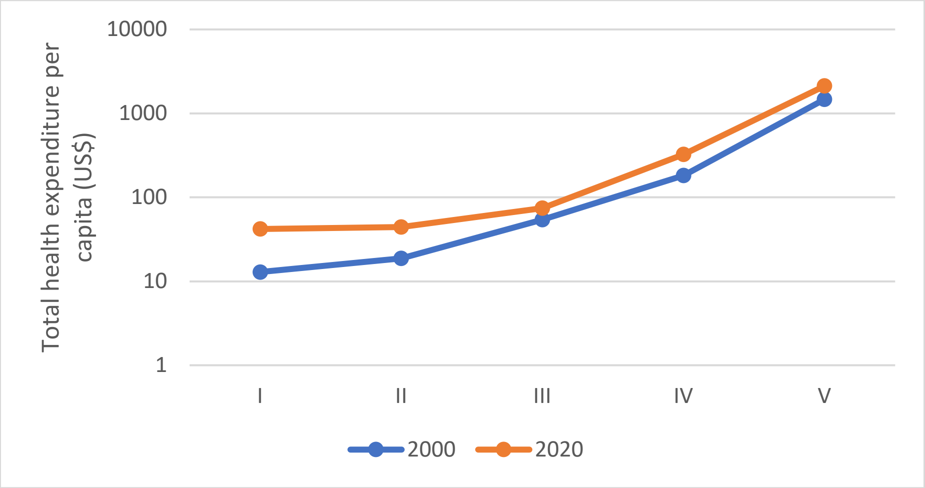

Figure G2 Total health expenditure as percentage of GDP, 2003, 2010, 2017, by transition phase

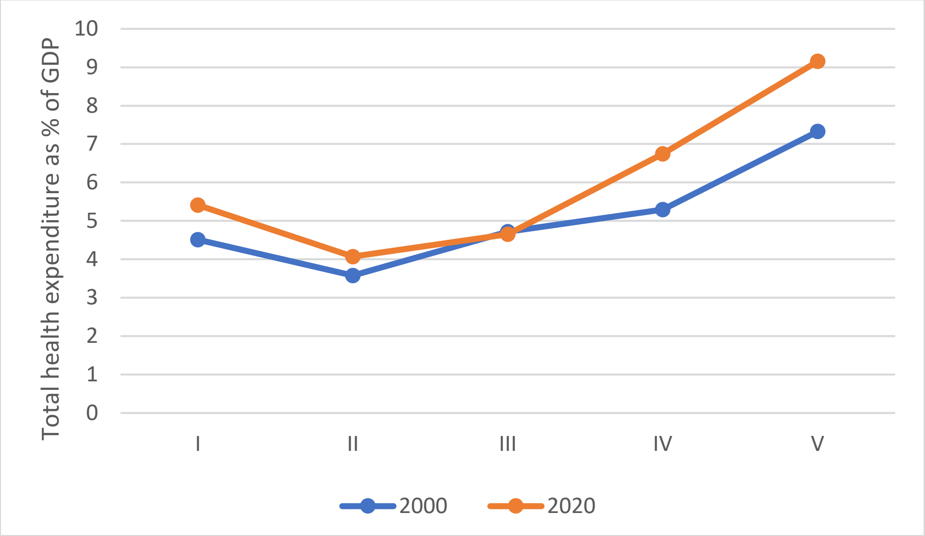

Figure G3 Out-of-pocket spending as percentage of total health expenditure, 2003, 2010, 2017, by transition phase

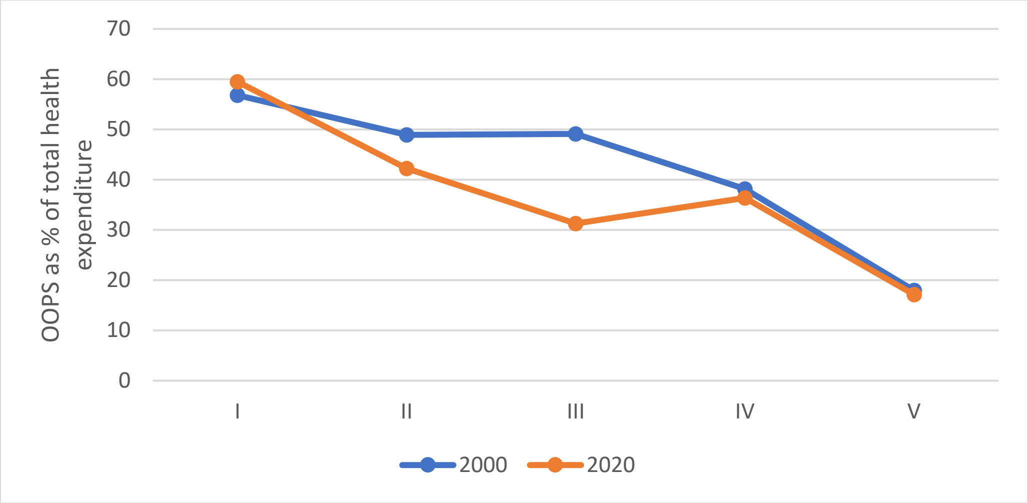

Health workforce

For health workforce we used data from WHO global databases to compute means for each country for 5-year period surrounding 2002 (2000-2004) and 2018 (2016-2020) to deal with missing values.[28] The density of core health professionals and skills mix ratio by phase are summarized in Figure G4 and G5 and Table G2. There is major increase in density especially in phase III and onward. The initial increase is predominantly driven by nurse-midwives, followed by a major increase in physicians and the skills mix ratio in phase III, but with major differences between 2002 and 2018.

Figure G4 Core health professionals (physicians, nurses, midwives) per 10,000 population, by transition phase and year

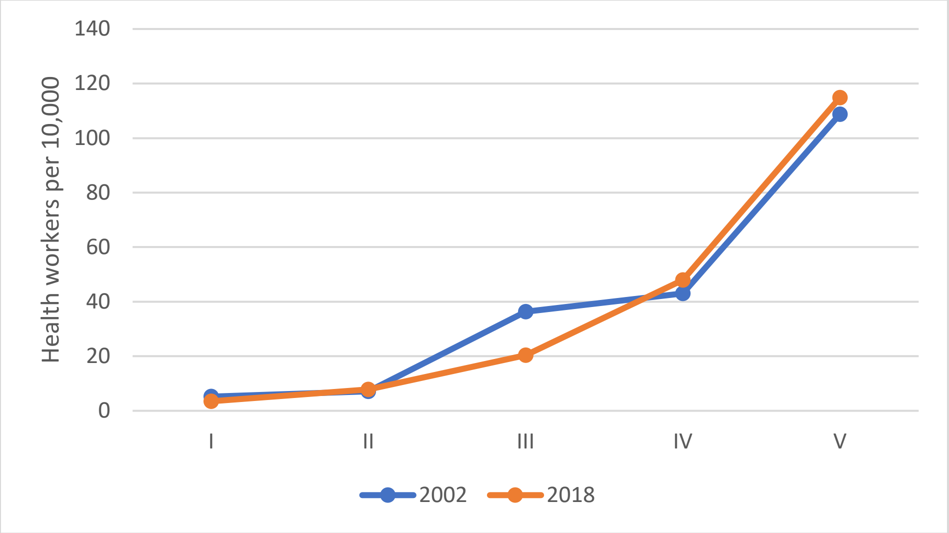

Figure G5. Nurse and midwife to physician ratio by transition phase and year

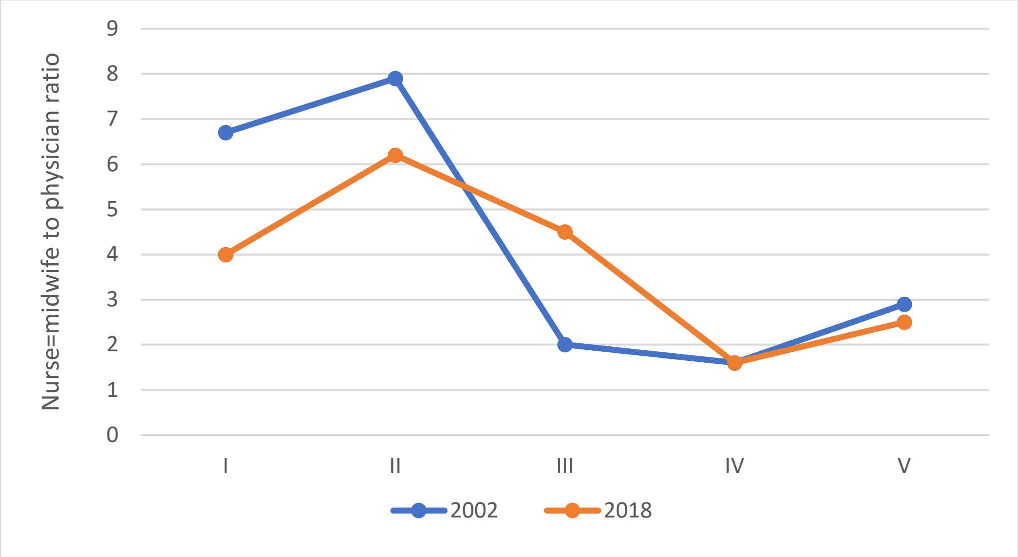

Table G2: Health workforce indicators by phase for 2002 (2000-2004) and 2018 (2016-2020): total core health professionals (physicians + nurse-midwives), physicians and nurse-midwives per 10,000 population and ratio nurse+midwives to physicians

		2002				2018			
Phase		Total health professionals per 10k	Physicians per 10k	Nurse-midwives per 10k	Ratio nurse midwife to physician	Total health professionals per 10k	Physicians per 10k	Nurse-midwives per 10k	Ratio nurse midwife to physician
I	Number	12	14	12	12	4	4	4	4
	Median	5.2	0.7	4.0	6.7	3.5	0.6	3.0	4.0
	P25	2.4	0.3	2.2	5.3	2.8	0.4	2.3	3.5
	P75	9.8	1.4	8.1	8.8	10.8	2.3	8.7	6.7
II	Number	27	30	28	27	25	25	25	25
	Median	7.1	1.4	6.5	7.9	7.8	1.2	6.1	6.2
	P25	5.4	0.5	4.6	4.2	5.3	0.8	4.2	3.0
	P75	10.9	2.1	9.5	11.5	12.2	1.9	9.1	8.3
III	Number	27	28	27	27	28	28	29	28
	Median	36.3	12.1	24.1	2.0	20.4	3.6	13.1	4.5
	P25	25.0	6.3	16.5	1.5	9.7	1.4	7.9	1.8
	P75	76.2	26.6	47.8	3.6	34.1	7.6	26.2	7.1
IV	Number	30	31	31	30	36	38	36	36
	Median	43.0	14.1	27.2	1.6	48.0	20.5	27.9	1.6
	P25	26.1	9.4	13.4	1.3	29.2	10.2	18.7	1.2
	P75	76.3	26.2	47.8	2.5	75.1	29.4	41.9	2.2
V	Number	30	32	33	30	46	48	47	46
	Median	108.8	28.0	75.5	2.9	114.9	34.2	79.8	2.5
	P25	76.7	22.5	49.8	2.1	93.5	26.4	60.4	1.8
	P75	127.4	33.6	96.0	3.9	146.1	41.5	111.3	3.3
Total		126	135	131	126	139	143	141	139
		35.2	12.2	24.0	2.7	41.8	16.6	29.0	2.6
		10.5	1.9	8.7	1.7	14.8	2.5	11.2	1.6
		84.9	26.2	55.9	5.7	98.1	33.1	62.2	4.4

Annex H: Service delivery coverage and inequalities

Figure H1: Median coverage of: antenatal care (first and fourth visit), institutional deliveries, and C-section rate per 100 live births by transition phase, DHS and MICS surveys, 2000-2020 (survey medians).

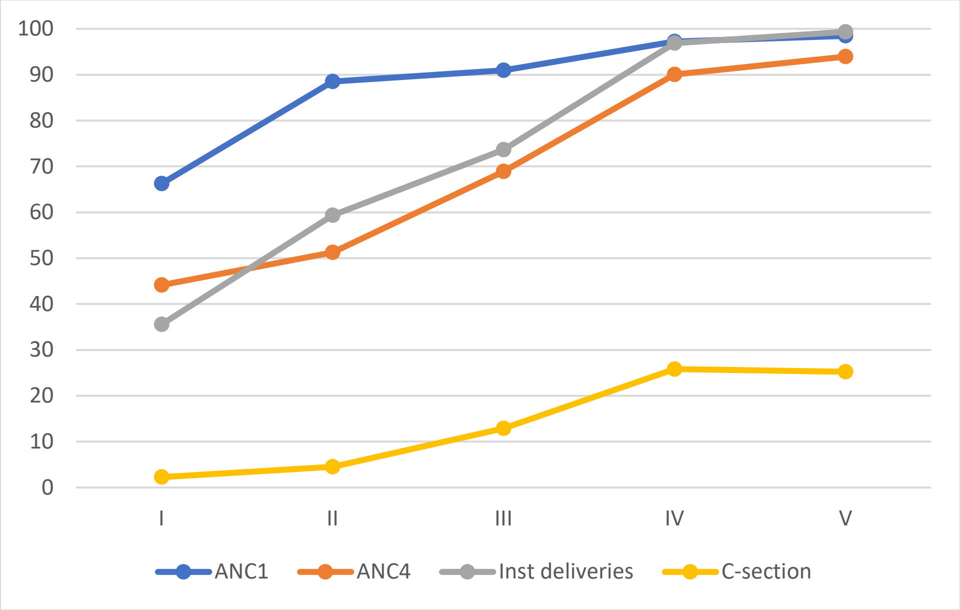

Figure H.2: Deliveries by level of facility (hospital/lower) by transition phase: country median, national DHS and MICS surveys (N=301), 2000-2020

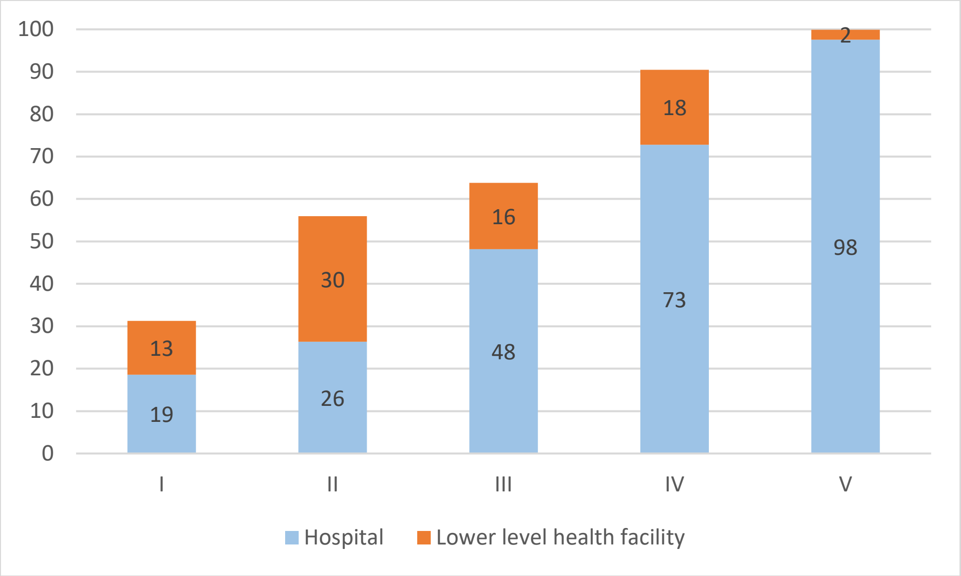

Table H3: Coverage of institutional live births by transition phase, national and among the poorest and richest wealth quintiles with absolute gaps and inequality pattern (N=324 national surveys, 2000-2020)

Phase	Number of surveys	National	Poorest	Richest	Absolute gap	Inequalities pattern	Description pattern
I	38	35.6	13.6	74.4	60.8	16.8	Top
II	126	59.4	35.2	91.5	56.3	8.0	Top
III	69	73.6	45.4	95.5	50.1	-6.4	Bottom
IV	76	96.9	91.8	98.9	7.2	-3.1	Bottom
V	15	99.3	99.1	99.7	0.6	0.1	None

*Inequality patterns are defined as the difference between the top plus bottom quintiles and the national mean.

Table H4: Caesarean sections per 100 live births by transition phase, national and among the poorest and richest wealth quintiles (N=287 surveys, 2000-2020)

Phase	Number of surveys	National	Poorest wealth quintile	Richest wealth quintile
I	32	2.3	0.8	7.4
II	111	4.5	1.7	10.5
III	62	12.9	4.3	27.9
IV	69	25.8	15.1	41.7
V	13	25.3	18.2	33.3
	287			

Annex I Summary table and percentiles for country assessments

Table I.1: Summary of maternal and peri-neonatal mortality transition phases and their characteristics

**Phases**	**I (highest mortality)**	**II**	**III**	**IV**	**V (lowest mortality)**
**Mortality levels**					
	~~I~~	~~II~~	~~III~~	~~IV~~	~~V~~
Maternal mortality per 100,000 live births	≥700	300-700	100-300	20-100	<20
Peri-neonatal mortality per 1,000 births (neonatal mortality)	≥80 (>45)	55-80 (30-45)	30-55 (15-30)	15-30 (<10-15)	<15 (< 10)
Ratio stillbirth + neonatal to maternal	<10	9-12	17-25	29-50	56-104
**Main causes of death (stillbirth, neonatal death, maternal death)**					
Group 1 (infections)	Major cause	Strong decline	Further decline	Less common	Uncommon
Group 2 (maternal/fetal / new-born health status)	Major cause	Limited decline, increasing importance	Limited decline, increasing importance	Decline, but major cause	Decline but main cause
Group 3 (peri-partum complications)	Major cause	Decline	Decline	Decline	Less common
**Fertility transition**					
Total fertility	Very high	High	Moderate	Moderate to low	Low
Adolescent fertility (15-19)	Very high	High	Moderate	Moderate to low	Low
Abortion policy	Most restrictive				Most permissive
**Health systems**					
Health financing	Low per capita expenditure, high OOPS	Increasing expenditure, low government share as % of GDP, high OOPS	Same as phase II, continuing	Same as phase III, continuing	Very high health spending, low OOP health expenditure, share of GDP
Health workforce (physicians, nurses, midwives)	Low density	Increase nurses midwives	Major increase density, skills mix ratio declining	Continued increase density, physicians driven	Core health workforce over 100 per 10,000 population
**Delivery of care strategies for transitions**					
Main challenges	Access	Access / quality	Access / quality	Quality	Quality & marginalized groups
Major interventions	Infection control; essential obstetric & neonatal care	+BEmONC, expanding CEmONC; demand generation	+BEmONC, full CEmONC	+ specialized care; small, sick newborn	+ universal access to CEmONC and to specialized care small sick newborn
Platform	Community, health faci-lities; ANC	Health centres, hospital; ANC	Health centres, more hospital; ANC	Health centers, more hospitals; ANC	Hospitals; ANC
Inequality pattern by wealth	Top / mass deprivation	Linear, steep slope	Bottom / marginalization of the poorest	Linear, small differences	Near-universal coverage
**Selected health indicators**					
Institutional delivery (IQR)	31 – 55%	39-77%	58-87%	92-99%	98% - 99.8%

Institutional delivery: rural (IQR)	24-43%	32-70%	45-75%	84-99%	98-99.7%
Institutional delivery: hospital (IQR)	14-30%	16-38%	32-68%	60-97%	84-99%
C-section: poorest (IQR)	0.5-1.5%	1.1-2.9%	2.3-9.9%	10.5-23.0%	14.2-33.4%
**Socio-economic**					
Female education, secondary enrollment (%), 2017	28.8	46.5	68.0	92.6	110.9
GNI per capita (US$), 2017	933	1056	3101	5654	28,300

The reference or typical values (median, 25th and 75th percentile) for the characteristics by phase of the maternal, stillbirth and neonatal mortality transition are derived from the analyses summarized in the paper. We used 2020 as the reference year for phases II to V. For phase I however there were only five countries in 2020. Therefore, we used 2000 as the reference year for this phase. It is noted that for all characteristics of the transition the medians and interquartile range were very similar in 2000 and 2020. The only exceptions were the socioeconomic indicators which had increased in all phases in comparison to the same phase in 2000. For the survey data derived values no data are given for phase V if there were less than 10 surveys.

Table I1: Indicators with mean, median, and percentile values (25th, 75th) by mortality transition phase.

**MORTALITY PATTERN**	**Phase**	**Median**	**p25**	**p75**
**Ratio (stillbirth+ neonatal mortality) to maternal death**	I	7.6	6.5	8.5
	II	10.3	9.4	12.0
	III	17.6	14.9	24.6
	IV	34.6	25.4	46.4
	V	84.3	55.1	118.7
**CAUSES OF NEONATAL DEATH**	**Phase**	**Median**	**p25**	**p75**
**Cause group 1 (% of all neonatal deaths)**	I	27.9	26.1	33.1
	II	19.8	18.5	22.6
	III	16.3	13.7	20.9
	IV	12.1	9.7	18.1
	V	7.3	4.8	9.9
**Cause group 2 (% of all neonatal deaths)**	I	43.9	39.1	45.8
	II	52.1	48.9	54.7
	III	57.7	53.0	61.1
	IV	69.6	65.0	72.9
	V	78.8	73.4	83.9
**Cause group 3 (% of all neonatal deaths)**	I	27.6	27.1	28.8
	II	27.8	27.4	28.8
	III	26.3	23.5	27.6
	IV	17.1	14.8	3.2
	V	14.7	9.2	17.7
**FERTILITY**	**Phase**	**Median**	**p25**	**p75**
**Total Fertility Rate**	I	6.1	5.8	6.9
	II	4.5	3.9	5.0
	III	3.1	2.3	4.3
	IV	2.1	1.7	2.8
	V	1.5	1.4	1.7
**ASFR (15-19)**	I	102	99	141

	Phase	Median	p25	p75
	II	97	32	23
	III	71	37	32
	IV	39	21	42
	V	11	9	49
**COVERAGE INTERVENTIONS**	**Phase**	**Median**	**p25**	**p75**
ANC 4 or more visits	I	44.1	18.1	56.3
	II	51.3	42.6	65.3
	III	68.9	55.0	81.1
	IV	90.0	84.1	94.3
	V	93.9	84.2	95.3
Institutional births	I	35.6	24.6	48.2
	II	59.4	42.6	74.9
	III	73.6	57.2	90.2
	IV	96.9	87.7	98.6
	V	99.3	98.6	99.8
C-sections	I	2.3	1.5	4.2
	II	4.5	3.0	6.4
	III	12.9	6.3	21.7
	IV	25.8	18.6	33.2
	V	25.3	18.4	29.6
**URBAN - RURAL INST BIRTH COVERAGE**	**Phase**	**Median**	**p25**	**p75**
Urban coverage	I	63.0	54.0	71.5
	II	84.5	77.8	92.4
	III	88.3	75.8	96.0
	IV	98.4	95.7	99.4
	V	99.6	98.8	99.9
Rural coverage	I	25.2	19.0	39.5
	II	47.9	32.8	68.4
	III	58.5	43.9	81.1
	IV	92.5	76.2	98.4
	V	99.1	98.3	99.7
Absolute difference urban - rural	I	35.7	29.6	40.7
	II	31.9	20.7	40.1
	III	24.1	13.6	34.5
	IV	5.9	0.6	20.5
	V	0.0	0.0	0.5
**POOR RICH INST BIRTH COVERAGE**	**Phase**	**Median**	**p25**	**p75**
Poorest quintile	I	13.6	9.5	24.8
	II	35.2	18.5	54.5
	III	45.4	26.4	72.5
	IV	91.8	69.0	96.9
	V	99.1	97.5	99.7
Richest quintile	I	74.4	57.4	82.9
	II	91.5	80.3	96.2
	III	95.5	90.7	98.2
	IV	98.9	97.8	99.6
	V	99.7	99.1	100.0
Absolute difference poor-rich	I	53.9	36.0	61.3
	II	51.0	37.8	61.9
	III	44.1	20.9	62.6
	IV	7.8	1.2	28.9
	V	0.2	-0.3	1.0
Pattern index poorest - richest	I	14.4	8.7	18.7

(poorest + richest - 2 * mean)	II	7.9	-1.0	15.8
	III	-0.7	-3.5	5.2
	IV	-2.6	-11.3	-1.1
	V	-0.1	-1.3	0.4
**POOR RICH C-SECTION RATES**	**Phase**	**Median**	**p25**	**p75**
Poorest quintile	I	0.8	0.3	1.7
	II	1.7	0.9	2.7
	III	4.3	2.1	10.7
	IV	15.1	7.4	21.9
	V	18.2	14.2	29.0
Richest quintile	I	7.4	4.4	9.8
	II	10.5	7.9	16.4
	III	27.9	14.4	47.3
	IV	41.7	30.8	54.9
	V	33.3	21.2	42.6
**POOR RICH NEONATAL MORTALITY RATES**	**Phase**	**Median**	**p25**	**p75**
Poorest quintile	I	40.7	32.6	58.38733
	II	34.3	28.7	40.09578
	III	27.5	23.2	33.14615
	IV	15.9	12.8	20.32477
	V			
Richest quintile	I	34.1	27.8	38.4
	II	26.7	22.1	31.6
	III	17.1	12.8	21.7
	IV	7.9	5.8	10.6
	V	8.5	0.4	16.7
**COVERAGE BIRTHS BY HOSPITALS**	**Phase**	**Median**	**p25**	**p75**
Coverage by hospitals	I	18.6	11.2	28.3
	II	26.3	15.8	33.9
	III	48.2	28.1	67.8
	IV	72.8	57.0	94.4
	V	97.6	92.3	98.7
Coverage by lower level facilities	I	12.7	6.0	17.6
	II	29.6	12.7	44.3
	III	15.7	3.1	31.2
	IV	17.7	3.0	30.9
	V	2.3	0.8	6.4
**SOCIO-ECONOMIC STATUS**	**Phase**	**Median**	**p25**	**p75**
GNI per capita (US $ constant)	I	280	200	400
	II	845	540	1420
	III	1825	1090	3405
	IV	4490	3350	7820
	V	22710	13905	44785
Secondary enrolment (gross), girls	I	16	12	24
	II	45	37	53
	III	74	47	80
	IV	98	89	103
	V	105	101	117
**HEALTH WORKFORCE DENSITY**	**Phase**	**Median**	**p25**	**p75**
Core health professionals per 10k	I	5.2	2.4	9.8
	II	7.8	5.3	12.2
	III	20.4	9.7	34.1
	IV	48.0	39.2	75.1

	V	114.8	93.5	146.1
Ratio nurse-midwife to physicians	I	6.7	5.3	8.8
	II	6.2	3.0	8.3
	III	4.5	1.8	7.1
	IV	1.6	1.2	2.2
	V	2.6	1.6	4.4
Physicians per 10k	I	0.7	0.3	1.4
	II	1.2	0.8	1.9
	III	3.6	1.4	7.6
	IV	20.5	10.2	29.4
	V	34.2	26.4	41.5
Nurse-midwives per 10k	I	4.0	2.2	8.1
	II	6.1	4.2	9.1
	III	13.1	7.9	26.2
	IV	27.9	18.7	41.9
	V	79.8	60.3	111.3
**NEONATAL MORTALITY BY PLACE OF DEL.**	**Phase**	**Median**	**p25**	**p75**
Neonatal mortality among home births	I	41.1	37.0	46.6
	II	32.6	28.3	37.0
	III	22.3	17.7	26.1
	IV	10.9	9.0	13.7
	V			
Neonatal mortality among hospital births	I	32.8	29.0	38.2
	II	26.1	21.3	31.8
	III	17.9	13.1	24.8
	IV	7.8	4.2	11.1
	V			
NMR among lower level facility births	I	31.3	26.1	34.1
	II	26.1	20.6	31.0
	III	14.5	8.2	22.6
	IV	9.8	4.5	12.5
	V			

[1] World Health Organization. Trends in maternal mortality 2000 to 2020: estimates by WHO, UNICEF, UNFPA, World Bank Group and UNDESA/Population Division. Geneva: WHO; 2023.

[2] United Nations Interagency Group for Child Mortality. Levels and trends in child mortality: report 2022. Estimates developed by the UN IGME. 2023.

[3] WHO review. National Center for Health Statistics. *Infant mortality problems in Norway* (Vital and Health Statistics, Analytical Studies, Series 3, No. 8). Washington, DC, United States Department of Health, Education and Welfare, 1967.

[4]National Center for Health Statistics. *Infant and perinatal mortality in Denmark* (Vital and Health Statistics, Analytical Studies, Series 3, No. 9). Washington, DC, United States Department of Health, Education and Welfare, 1967.

[5] National Center for Health Statistics. *Infant loss in the Netherlands*. Washington, DC, United States Department of Health, Education and Welfare (Vital and Health Statistics, Analytical Studies, Series 3, No. 11), 1968.

[6] National Center for Health Statistics. *Infant and perinatal mortality in England and Wales* (Vital and Health Statistics, Analytical Studies, Series 3, No. 12). Washington, DC, United States Department of Health, Education and Welfare, 1968.

[7] Goudar SS, Goco N, Somannavar MS, et al. Institutional deliveries and stillbirth and neonatal mortality in the Global Network's Maternal and Newborn Health Registry. Reprod Health. 2020;17(Suppl 3):179.

[8] Alliance for Maternal and Newborn Health Improvement (AMANHI) mortality study group. Population-based rates, timing, and causes of maternal deaths, stillbirths, and neonatal deaths in south Asia and sub-Saharan Africa: a multi-country prospective cohort study. Lancet Glob Health. 2018;6(12):e1297-e1308.

[9] Colbourn T, Nambiar B, Bondo A, Makwenda C, Tsetekani E, Makonda-Ridley A, et al. Effects of quality improvement in health facilities and community mobilization through women's groups on maternal, neonatal and perinatal mortality in three districts of Malawi: MaiKhanda, a cluster randomized controlled effectiveness trial. Int Health. 2013 Sep;5(3):180-95.

[10] Anwar J, Torvaldsen S, Sheikh M, Taylor R. Completeness of a Maternal and Perinatal Mortality Enhanced Surveillance System in Pakistan: Evidence from Capture-Recapture Methods. Matern Child Health J. 2018 Dec;22(12):1743-1750

[11] Hug L, You D, Blencowe H, Mishra A, Wang Z, Fix MJ, Wakefield J, et al. Global, regional, and national estimates and trends in stillbirths from 2000 to 2019: a systematic assessment. Lancet. 2021;398(10302):772-785.

[12] Goudar SS, Goco N, Somannavar MS, et al. Institutional deliveries and stillbirth and neonatal mortality in the Global Network's Maternal and Newborn Health Registry. Reprod Health. 2020;17(Suppl 3):179.

[13] Alliance for Maternal and Newborn Health Improvement (AMANHI) mortality study group. Population-based rates, timing, and causes of maternal deaths, stillbirths, and neonatal deaths in south Asia and sub-Saharan Africa: a multi-country prospective cohort study. Lancet Glob Health. 2018;6(12):e1297-e1308.

[14] Say L, Chou D, Gemmill A, Tunçalp Ö, et al. Global causes of maternal death: a WHO systematic analysis. Lancet Glob Health. 2014 Jun;2(6):e323-33. The leading causes of maternal death groups are infections, hemorrhage, hypertensive disorders, abortion complications, and non-obstetric or indirect causes.

[15] Lawn JE, Blencowe H, Waiswa P, et al.; Lancet Ending Preventable Stillbirths Series study group; Lancet Stillbirth Epidemiology investigator group. Stillbirths: rates, risk factors, and acceleration towards 2030. Lancet. 2016 Feb 6;387(10018):587-603.

[16] Perin J, Mulick A, Yeung D, Villavicencio F, Lopez G, Strong KL, et al. Global, regional, and national causes of under-5 mortality in 2000-19: an updated systematic analysis with implications for the Sustainable Development Goals. Lancet Child Adolesc Health 2022;6(2):106-115.

[17] De Brouwere V, Tonglet R, Van Lerberghe W. Strategies for reducing maternal mortality in developing countries: what can we learn from the history of the industrialized West? Trop Med Int Health 1998; 3(10): 771-82.

[18] Loudon I. Deaths in childbed from the eighteenth century to 1935. Med Hist. 1986 Jan;30(1):1-41.

[19] Chamberlain G. British maternal mortality in the 19th and early 20th centuries. J R Soc Med. 2006 Nov;99(11):559-63.

[20] WB report

[21] Loudon I. Maternal mortality in the past and its relevance to developing countries today. Am J Clin Nutr. 2000 Jul;72(1 Suppl):241S-246S.

[22] McCaw-Binns AM, Campbell LV, Spence SS. The evolving contribution of non-communicable diseases to maternal mortality in Jamaica, 1998-2015: a population-based study. BJOG. 2018 Sep;125(10):1254-1261.

[23] Say L, Chou D, Gemmill A, Tunçalp Ö, et al. Global causes of maternal death: a WHO systematic analysis. Lancet Glob Health. 2014 Jun;2(6):e323-33.

[24]Graham W, Woodd S, Byass P, et al. Diversity and divergence: the dynamic burden of poor maternal health. Lancet 2016;388(10056):2164-2175.

[25] Kassebaum NJ, Bertozzi-Villa A, Coggeshall MS, et al. Global, regional, and national levels and causes of maternal mortality during 1990-2013: a systematic analysis for the Global Burden of Disease Study 2013. Lancet. 2014 Sep 13;384(9947):980-1004.

[26] Perin J, Mulick A, Yeung D, et al. Global, regional, and national causes of under-5 mortality in 2000-19: an updated systematic analysis with implications for the Sustainable Development Goals. Lancet Child Adolesc Health. 2022 Feb;6(2):106-115. doi: 10.1016/S2352-4642(21)00311-4.

[27] WHO Global Health Expenditure database (GHED). https://apps.who.int/nha/database/Select/Indicators/en

[28] WHO global health observatory. https://www.who.int/data/gho/data/themes/topics/health-workforce
